# Mechanistic model of nutrient uptake explains dichotomy between marine oligotrophic and copiotrophic bacteria

**DOI:** 10.1371/journal.pcbi.1009023

**Published:** 2021-05-19

**Authors:** Noele Norris, Naomi M. Levine, Vicente I. Fernandez, Roman Stocker

**Affiliations:** 1 Department of Electrical Engineering and Computer Science, Massachusetts Institute of Technology, Cambridge, United States of America; 2 Department of Biological Sciences, University of Southern California, Los Angeles, United States of America; 3 Institute of Environmental Engineering, Department of Civil, Environmental and Geomatic Engineering, ETH Zürich, Zürich, Switzerland; University of Connecticut School of Medicine, UNITED STATES

## Abstract

Marine bacterial diversity is immense and believed to be driven in part by trade-offs in metabolic strategies. Here we consider heterotrophs that rely on organic carbon as an energy source and present a molecular-level model of cell metabolism that explains the dichotomy between copiotrophs—which dominate in carbon-rich environments—and oligotrophs—which dominate in carbon-poor environments—as the consequence of trade-offs between nutrient transport systems. While prototypical copiotrophs, like *Vibrios*, possess numerous phosphotransferase systems (PTS), prototypical oligotrophs, such as SAR11, lack PTS and rely on ATP-binding cassette (ABC) transporters, which use binding proteins. We develop models of both transport systems and use them in proteome allocation problems to predict the optimal nutrient uptake and metabolic strategy as a function of carbon availability. We derive a Michaelis–Menten approximation of ABC transport, analytically demonstrating how the half-saturation concentration is a function of binding protein abundance. We predict that oligotrophs can attain nanomolar half-saturation concentrations using binding proteins with only micromolar dissociation constants and while closely matching transport and metabolic capacities. However, our model predicts that this requires large periplasms and that the slow diffusion of the binding proteins limits uptake. Thus, binding proteins are critical for oligotrophic survival yet severely constrain growth rates. We propose that this trade-off fundamentally shaped the divergent evolution of oligotrophs and copiotrophs.

## Author summary

Marine bacteria utilize carbon as a building block and an energy source and thus exert an important control on the amount of carbon that is sequestered in the ocean versus respired into the atmosphere. They use a spectrum of strategies to consume carbon: while copiotrophic bacteria dominate in nutrient-rich environments, oligotrophic bacteria dominate in nutrient-poor environments and are typically smaller, nonmotile, and slower growing. Yet the paragon oligotroph SAR11 is the planet’s most abundant organism. Despite this, most of our understanding of bacteria derives from research on copiotrophs. Here we use molecular-level models to understand how an oligotroph’s physiology enables it to outperform copiotrophs in nutrient-poor but not in nutrient-rich environments. We contrast copiotrophs’ prevalent method of sugar transport with oligotrophs’ reliance on binding proteins, which trap nutrients in the periplasm. Binding proteins allow cells to attain affinities that are much higher than the transport proteins’ intrinsic affinities. However, our model predicts that attaining such high affinities requires large periplasms with high abundances of the slowly diffusing binding proteins, which precludes high growth rates. By quantifying the benefits and costs of binding proteins, we provide a mechanistic explanation for the divergent evolution of oligotrophs and copiotrophs.

## Introduction

Approximately half of global carbon fixation occurs in the ocean [1]. The fate of that carbon is governed by diverse species of heterotrophic bacteria [2–4] that differ in their carbon preferences and uptake rates [5–7]. Yet we lack a fundamental understanding of how and why species’ metabolic strategies differ, an understanding needed to predict how a changing climate will affect rates of carbon flux in the ocean [8].

An important driver of species’ differentiation is nutrient availability, leading to a spectrum of microbial lifestyles: at opposite ends, copiotrophs dominate in nutrient-rich environments, whereas oligotrophs dominate in nutrient-poor environments [9–11]. Prototypical copiotrophs, like *Vibrios*, exhibit a feast-and-famine lifestyle and swim to colonize sporadic, nutrient-rich patches and particles [12,13]. They reach volumes greater than 1 μm^3^ and doubling times less than one hour [14]. Conversely, the abundant oligotrophs of the SAR11 clade are nonmotile and free-living [15] and have volumes smaller than 0.1 μm^3^ and doubling times greater than 5 hours [14]. Although copiotrophs typically attain higher doubling rates and have larger per cell biomass, the slow-growing oligotrophs comprise the majority of marine bacterial biomass [16,17]. Despite this, most of our understanding of bacterial metabolism derives from research on copiotrophic-like bacteria, which are easier to culture [18].

Genomic analyses suggest that the divergent phenotypic traits of copiotrophs and oligotrophs are correlated with their suite of genes for nutrient transport [14,19–21]. Prototypical copiotrophs have many genes for phosphotransferase systems (PTS) used to uptake specific sugars [14,22]. In contrast, prototypical oligotrophs, like SAR11 and *Sphingopyxis alaskensis*, lack PTS [14,23] and instead rely heavily on ATP-binding cassette (ABC) transport systems, which are comprised of a transmembrane transport unit and a periplasmic substrate-binding protein. ABC transport systems have higher affinities than PTS [24,25]. Although it has long been held that the high affinity of ABC transport is a consequence of high-affinity binding proteins [26,27], Bosdriesz and others recently suggested that the affinity of ABC transport is a function of binding protein abundance and, specifically, that ABC transport confers high affinity only when the abundance of binding proteins exceeds that of transport units [28]. Thus, oligotrophs’ high abundances of binding proteins may explain their ability to grow in low nutrient conditions [19,29]. However, it is not understood why oligotrophs such as SAR11 cannot achieve higher grower rates in nutrient-rich conditions or why typical copiotrophs—which do, in fact, possess many ABC transport systems—cannot achieve higher affinities in nutrient-limited conditions [9].

To understand the metabolic constraints governing the dichotomy between the oligotrophic and copiotrophic lifestyles, we develop molecular-level transport and cellular proteome allocation models to compare the performance of ABC transport and PTS. We derive a Michaelis–Menten approximation of ABC transport kinetics that predicts that the specific affinity of transport is proportional to binding protein abundance when the binding protein to transport unit ratio is sufficiently high. We thus find that ABC transport allows independent tuning of affinity and maximal uptake rate so that cells can achieve high affinities while closely matching transport and metabolic capacities. We thus predict that an oligotroph can attain a half-saturation concentration over a thousand-fold smaller than its binding protein’s dissociation constant. However, attaining this high affinity requires a great abundance of binding proteins, which diffuse slowly and require large periplasms. Consequently, the reliance on binding proteins to achieve high affinity precludes high growth rates. Moreover, the ability of ABC transport to achieve high affinities while matching metabolic capacity makes metabolic imbalances unlikely and thus mechanisms for handling sudden nutrient up-shifts typically unnecessary, which may explain the toxicity of high-nutrient conditions to SAR11. Together, these findings provide a mechanistic explanation for the divergence of the copiotrophic and oligotrophic lifestyles, as the consequence of trade-offs between PTS and ABC transport.

## Results

### The specific affinity of ABC transport is a function of both transport and binding protein abundance

**Fig 1:**
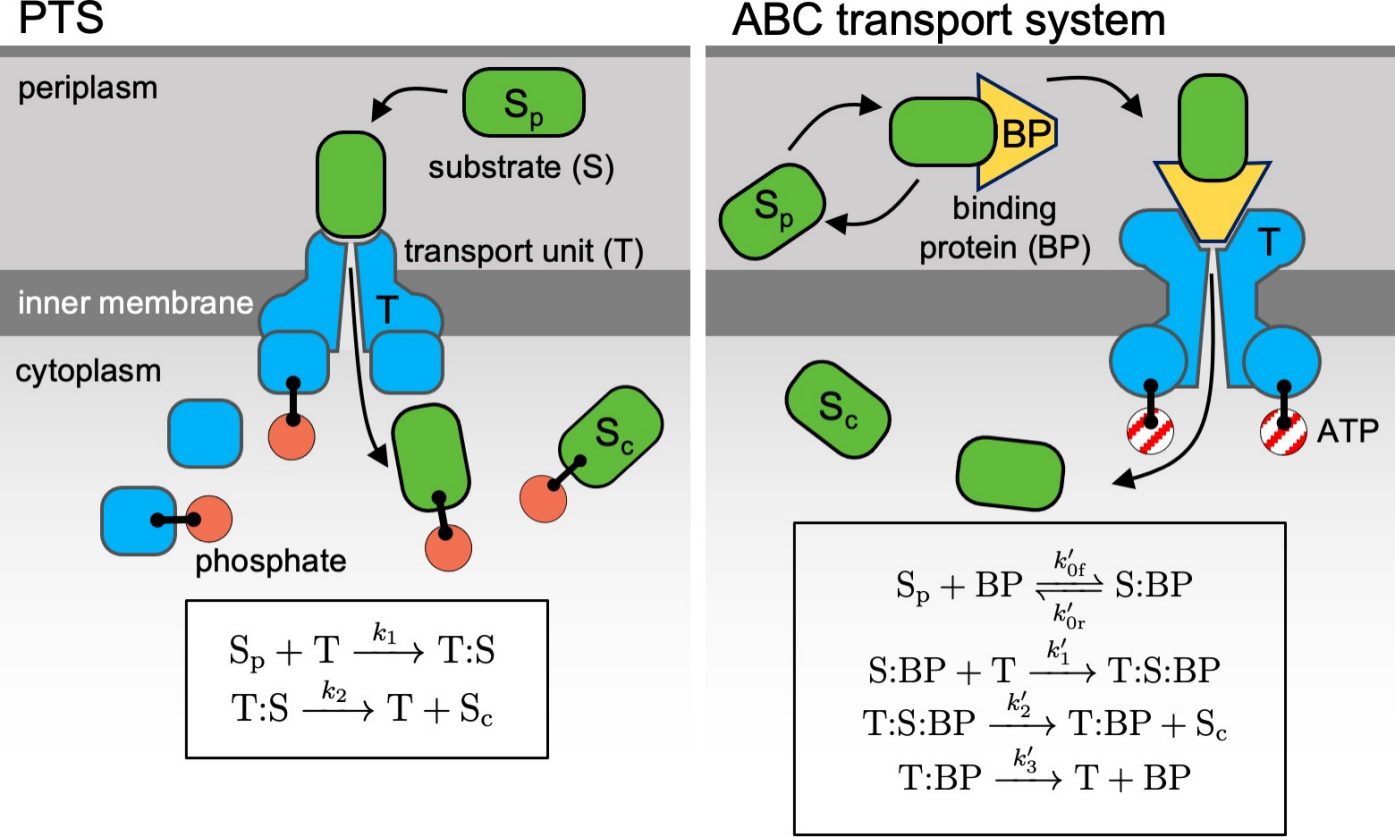
Schematic of transport systems. For a nutrient to enter the cytoplasm, a transport unit bound to the inner membrane must expend energy to modify the substrate or translocate the substrate against a concentration gradient. For transport of a sugar by a phosphotransferase system (PTS), the sugar binds directly to the transport unit, and a cascade of specific proteins phosphorylate that particular sugar. For transport of a substrate by an ATP-binding cassette (ABC) transport system, binding proteins in the periplasm first scavenge for and store the substrate in the periplasm. When bound to substrate, a binding protein can then bind to a membrane-bound transport unit, which uses ATP to translocate the substrate. While a single type of binding protein may be able to bind to different substrates, it can bind to only a single, corresponding type of transport unit. To limit the number of free parameters when modeling these two transport systems, we use a simple model of PTS that assumes that binding of the substrate to the transport unit is irreversible. We extend the model for ABC transport to account for the reversible binding of the substrate to the binding protein and the dissociation of the binding protein from the transport unit after translocation.

**Fig 2:**
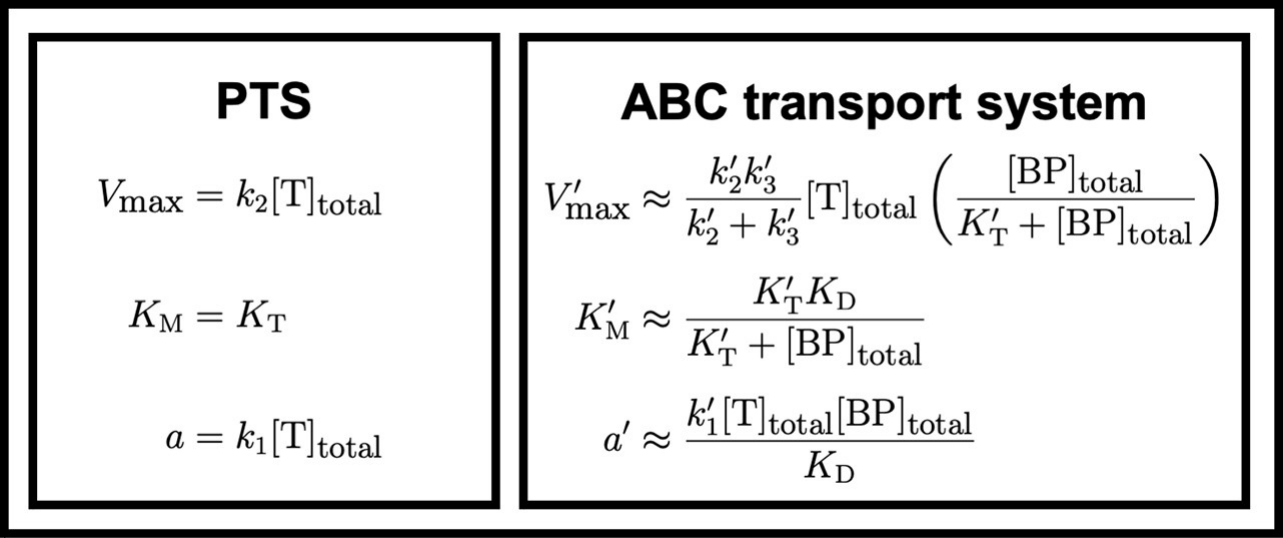
Maximal uptake rates, half-saturation concentrations, and specific affinities of PTS and ABC transport systems. We can approximate cytoplasmic uptake rates using the Michaelis–Menten equation: *v*_c_ = *V*_max_[S]_p_/(*K*_M_+[S]_p_), where *V*_max_ is the maximal uptake rate and *K*_M_ the half-saturation concentration. While the exact solution of the cytoplasmic uptake rate for our model of PTS is in the form of a Michaelis–Menten equation, the exact solution of the uptake rate for ABC transport is not. Because our simulations suggest that the abundance of binding proteins should exceed the abundance of transport units in the oligotrophic conditions where ABC transport is optimal, we make the approximations that (*i*) [T:S:BP]+[T:BP]≪[BP]_total_ and (*ii*) *k*′_1_[T]≪*k*_0r_ (Section B in S1 Appendix) to obtain the above estimates for the effective maximal rate and half-saturation concentration. For PTS, the half-saturation concentration is a constant equal to the dissociation constant *K*_T_ = *k*_2_/*k*_1_. For ABC transport, the half-saturation concentration depends on both the transport dissociation constant ${K^{\prime }}_{T}={k^{\prime }}_{2}k_{3}^{\prime }/\left({k^{\prime }}_{1}(k_{2}^{\prime }+k_{3}^{\prime })\right)$ and the binding protein dissociation constant *K*_D_ = *k*′_0r_/*k*′_0f_ and is additionally a function of the abundance of binding proteins. Under this approximation, the specific affinity *a*′ = *V*′_max_/*K*′_M_ of ABC transport is thus proportional to the product of the abundances of transport units and of binding proteins.

To contrast the nutrient acquisition strategies of PTS and ABC transport systems, we present models of both, which show that, whereas the half-saturation concentration of PTS is an intrinsic property of the transporter, the half-saturation concentration of ABC transport is a function of binding protein abundance [28]. A PTS is used for the cytoplasmic uptake of a specific sugar and modifies the sugar once it enters the cytoplasm by binding the sugar to a phosphate group. PTS uptake kinetics can be described by the canonical model for transport [30]. It describes transport as a two-step reaction, in which (*i*) the periplasmic substrate (S_p_) binds to the membrane-bound transport unit (T) with rate constant *k*_1_ to form a bound complex (T:S), and (*ii*) the substrate is translocated irreversibly into the cytoplasm with rate *k*_2_ (S_c_) (Fig 1 and Section A in S1 Appendix). Using mass-action kinetics, we find that the cytoplasmic uptake rate (the rate at which S_p_ is converted to S_c_) for PTS at steady-state is

$$v_{c,PTS}=k_{2}[T:S]=k_{2}{\left[T\right]}_{total}\frac{{\left[S\right]}_{p}}{K_{T}+{\left[S\right]}_{p}},$$ (1)

where *K*_T_ = *k*_2_/*k*_1_ is the transport unit dissociation constant and [T]_total_ is the abundance of membrane-bound transport units divided by the volume of the periplasm. (Note that we here express all transport rates in terms of change in periplasmic concentration per time. We use the conversion factor f_p_/(1−f_p_) to obtain the uptake rate in terms of change in cytoplasmic concentration, where f_p_ is the fraction of the cell’s volume comprised of the periplasm. See Section D.3 in S1 Appendix.) The solution in Eq 1 has the classic Michaelis–Menten form of nutrient transport [31], with maximal uptake rate *V*_max_ proportional to [T]_total_ and half-saturation constant *K*_M_ equal to *K*_T_ (Fig 2).

In contrast to PTS, the kinetics of ABC transport does not follow the classic Michaelis–Menten form [28]. ABC transport uses binding proteins (BP) in the periplasm that scavenge for incoming nutrients. These binding proteins, when in complex with the substrate, bind to membrane-bound transport units that require ATP to translocate the substrate from the periplasm into the cytoplasm [32–34]. Similar to previous models of transport by binding proteins [27,28], we describe ABC uptake by extending the PTS model to account for a four-step reaction: (*i*) the substrate–binding protein complex (S:BP) is formed by a reversible reaction with association rate *k*′_0f_ and dissociation rate *k*′_0r_, (*ii*) the bound complex of substrate and binding protein (S:BP) binds with rate constant *k*′_1_ to the membrane-bound transport unit (T) to form a bound complex (T:S:BP), (*iii*) the substrate is translocated irreversibly into the cytoplasm (S_c_) with rate *k*′_2_, and (*iv)* the transport unit and binding protein dissociate with rate *k*′_3_ (Fig 1). At steady state, we obtain a system of four equations that can be solved exactly for the cytoplasmic uptake rate for ABC transport, *v*_c,ABC_, in terms of change in periplasmic concentration per time as a function of the concentration of free substrate in the periplasm, [S]_p_ (Sections A.2 and D.3.2 in S1 Appendix):

$$v_{c,ABC}={k^{\prime }}_{2}\left(\frac{k_{3}^{\prime }}{k_{2}^{\prime }+k_{3}^{\prime }}\right){\left[T\right]}_{total}\frac{\left[S:BP\right]}{{K^{\prime }}_{T}+[S:BP]},\;{K^{\prime }}_{T}=\frac{{k^{\prime }}_{2}k_{3}^{\prime }}{{k^{\prime }}_{1}(k_{2}^{\prime }+k_{3}^{\prime })},$$ (2)

$$[S:BP]=\frac{{\left[S\right]}_{p}[BP]}{K_{D}+{k^{\prime }}_{1}[T]/{k^{\prime }}_{0f}},\;K_{D}=\frac{{k^{\prime }}_{0r}}{{k^{\prime }}_{0f}},$$ (3)

$$[BP]={\left[BP\right]}_{total}-[S:BP]-(1+k_{2}^{\prime }/k_{3}^{\prime })[T:S:BP],$$ (4)

$$[T]={\left[T\right]}_{total}-(1+k_{2}^{\prime }/k_{3}^{\prime })[T:S:BP].$$ (5)

This model is a simplification of the ABC transport model developed by Bosdriesz and others [28]; in contrast to their model, our model assumes that translocation as well as the association and dissociation of binding protein and transport unit proceed irreversibly and thus has three fewer free parameters. Yet we find that our model provides a good fit for the well-characterized maltose ABC transport system in *Escherichia coli* (Section C in S1 Appendix). The model accurately predicts the observed *K_M_* as well as the shape of the uptake rate curves as functions of both extracellular maltose concentration and binding protein abundance (Figs A-D in S1 Appendix).

To obtain a compact analytical expression describing how transport protein abundances affect uptake rate, we used our model to derive an approximation of ABC transport kinetics in Michaelis–Menten form. By assuming that binding proteins are much more abundant than active transport units [28,35] ([BP]_total_≫[T:S:BP]+[T:BP]) and that the abundance of unbound transport units is low (so that [T]≪*k*′_0r_/*k*′_1_) (Fig 1 and Section B in S1 Appendix), we obtain from Eqs 2–5 the following approximation for the cytoplasmic uptake rate:

$$v_{ABC}\approx \frac{k_{2}^{\prime }k_{3}^{\prime }}{k_{2}^{\prime }+k_{3}^{\prime }}{\left[T\right]}_{total}\left(\frac{{\left[BP\right]}_{total}}{K_{T}^{\prime }+{\left[BP\right]}_{total}}\right)\frac{{\left[S\right]}_{p}}{\frac{K_{T}^{\prime }K_{D}}{K_{T}^{\prime }+{\left[BP\right]}_{total}}+{\left[S\right]}_{p}}.$$ (6)

This Michaelis–Menten equation well approximates ABC transport when the binding protein to transport unit ratio sufficiently exceeds one and thus captures the dynamics of the full ABC transport model (Eqs 2–5) over a wide range of parameter values (S1 Fig).

This formulation shows analytically how the half-saturation “constant” *K*_M_ is, in fact, a function of the concentration of binding proteins in the periplasm ([BP]_total_, Fig 2). For ${{\left[BP\right]}_{total}\gg {\left[T\right]}_{total}\;and\;[BP]}_{total}\gg K_{T}^{\prime }$, as is the case for *E*. *coli*’s ABC maltose transport system, the approximation predicts that the half-saturation concentration *K*_M_ is proportional to both the transport dissociation constant $K_{T}^{\prime }$ and the binding protein dissociation constant *K*_D_ and is inversely proportional to the total abundance of binding proteins [BP]_total_. Therefore, expressing high abundances of binding proteins enables oligotrophs to attain small *K*_M_ values and thus high affinities. At low nutrient concentrations, the classic Michaelis–Menten uptake rate is proportional to the specific affinity [36], *a* = *V*_max_/*K*_M_. Whereas a bacterium using PTS has constant *K*_M_ and thus can increase its specific affinity in oligotrophic conditions only by tuning *V*_max_ (via expression of the transport unit; Eq 1), a bacterium using ABC transport can increase its specific affinity by tuning either *V*_max_ or *K*_M_, by tuning the expression levels of the transport units and binding proteins, respectively (Eq 6).

### A rate–affinity trade-off drives the differentiation of oligotrophs and copiotrophs

**Fig 3:**
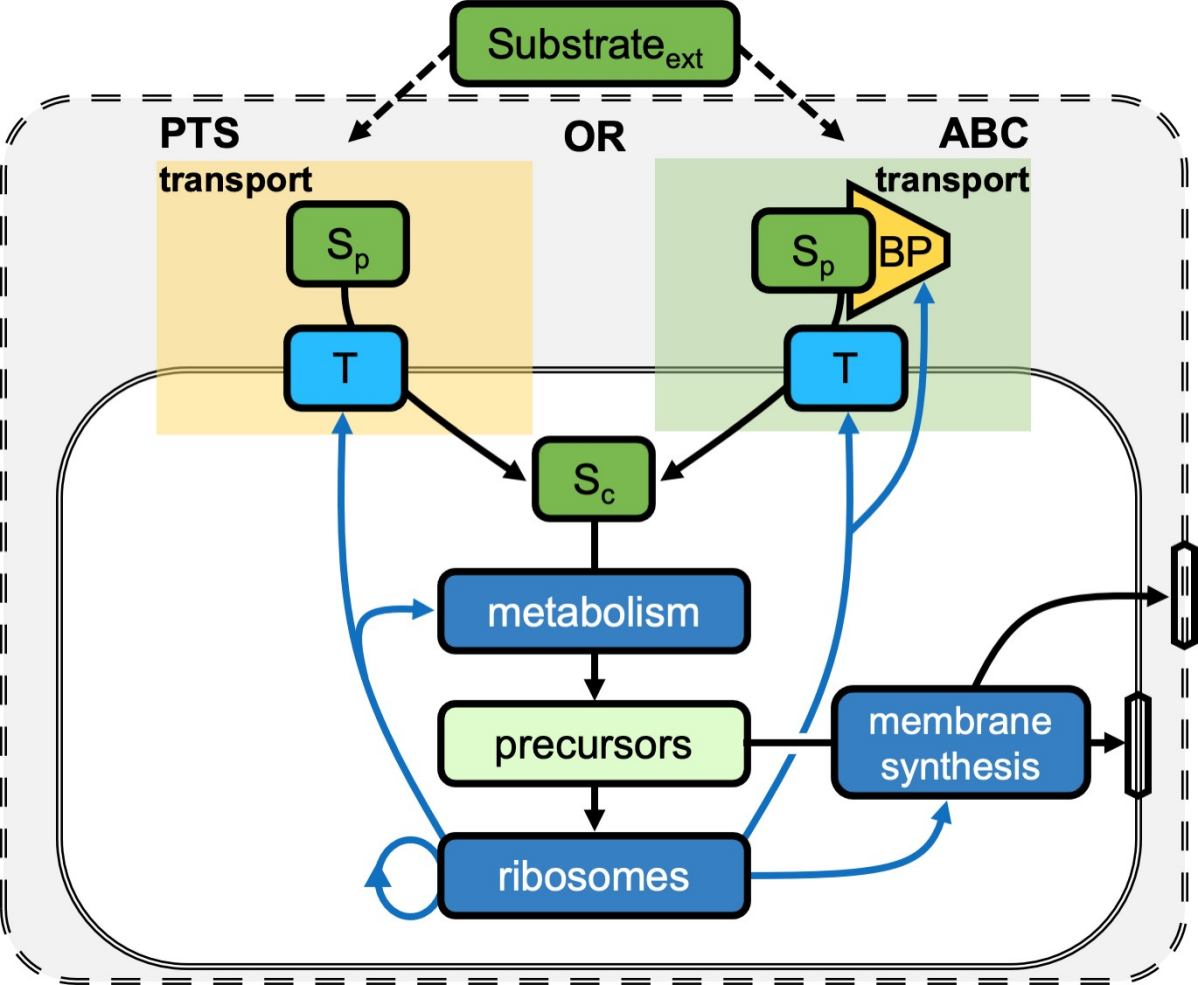
A simple metabolic model tracks the utilization of a generic nutrient by the cell. The nutrient diffuses into the periplasm via a porous outer membrane and is then transported into the cytoplasm by membrane-bound transport units. The cell uses either transport by PTS, in which the substrate directly binds to the transport unit, or ABC transport, in which the substrate must first bind to a binding protein and then this complex binds to the transport unit. The intracellular substrate is next metabolized by a protein group that transforms the substrate into a precursor (a generic amino acid) that is needed to build the cell. The precursors are used (*i*) by a membrane biosynthesis protein group to build both the outer and inner membranes and (*ii*) by ribosomes to make proteins comprising the six protein groups. This model is subject to a number of constraints to determine the proteome allocation that maximizes the steady-state exponential growth rate. While this model does not consider the utilization of carbon for energy, we expanded the model to consider energy to show that differences in the energetic requirements of PTS and ABC transport do not change our results (Section E in S1 Appendix).

The derived Michaelis–Menten kinetics (Eqs 1 and 6) show how ABC transport systems allow bacteria to achieve higher substrate affinities than PTS by expressing high abundances of binding proteins. To understand the costs associated with achieving these high affinities and thus to determine how the optimal expression levels of transport units and binding proteins differ in low-nutrient and high-nutrient environments, we integrate our solutions for the cytoplasmic uptake rates of PTS (Eq 1) and ABC transport (Eqs 2–5) into a mechanistic, single-cell metabolic model (Fig 3, Methods, and Section D in S1 Appendix). Similar to the self-replicator model of Molenaar and others [37], our highly idealized metabolic model accounts for only four protein groups–transport proteins, metabolic proteins, ribosomes, and membrane biosynthesis proteins–and is used to solve a proteome allocation problem that determines the optimal amount of each protein group that the cell should express in order to maximize its growth rate for a given extracellular nutrient concentration.

Our metabolic model tracks the transport of a nutrient into the cytoplasm and the subsequent transformation of that nutrient into the proteins and metabolites required for replication. The abundances of proteins and metabolites are constrained by the cell’s surface-area-to-volume ratio. Because the cellular components occupy volume, they are limited by maximum cytoplasmic and periplasmic densities to prevent molecular overcrowding [37], and this favors smaller surface-area-to-volume ratios. On the other hand, the surface of the inner membrane must be sufficiently large because the membrane-bound transport units carry “real estate costs” [37,38]. Larger surface-area-to-volume ratios also support higher specific uptake rates by diffusion at low-nutrient conditions [39–41]. Thus, taken together, the surface-area-to-volume ratio creates a trade-off between the cell’s capacity for uptake and its capacity for synthesis. Therefore, in addition to determining the optimal proteome allocations, our model also determines the optimal surface-area-to-volume ratio, the protein and metabolite concentrations that are constrained by this ratio, and the fraction of the volume devoted to the periplasm (Methods, Section D in S1 Appendix).

Central to this optimization problem are the costs and benefits of expressing more of a particular protein group. While expressing more transport units or binding proteins increases the uptake rate, it incurs a proteomic cost [35,42–44]. This cost is an opportunity cost. For example, because growth rate depends on the proteome fraction allocated to ribosomes [45], expressing greater abundances of transport proteins may limit growth, as it limits the proportion of the proteome devoted to ribosomes. We assume that the transport units of PTS and ABC transport systems have the same proteomic cost and that the proteomic cost of an ABC binding protein is four times less than the cost of a transport unit (see Section D.3 in S1 Appendix for justifications).

The effect that these transport proteomic costs have on the optimal proteome allocation strongly depends on the uptake rate per transport unit. This uptake rate is often limited by rates of diffusion within the periplasm [46,47]. Hence, we argue that differences in substrate diffusion drive a trade-off between PTS and ABC transport. Because ABC binding proteins are much larger than the substrates they bind, the diffusivity of the binding proteins is lower than the diffusivity of the substrate, limiting the achievable rates of ABC transport relative to PTS, as suggested by [28]. For example, a typical binding protein (MalE) has a molecular weight of approximately 40 kDa and thus an estimated cytoplasmic diffusivity of 2 μm^2^/s, whereas glucose has a molecular weight of 0.18 kDa and thus an estimated cytoplasmic diffusivity of 200 μm^2^/s [48]. Our model therefore assumes that the association rate *k*′_1_(Fig 1) is one-hundred times smaller than the equivalent rate for PTS (i.e., *k*′_1_ = 0.01*k*_1_) because it depends on the slow diffusion of the binding protein toward the membrane-bound transport units.

We choose the other rate values to enable a fair comparison between PTS and ABC transport. We assume that both their translocation rates and transport unit dissociation constants are equal (*k*′_2_ = *k*_2_, *K*′_T_ = *K*_T_). Therefore, since *K*_T_ = *k*_2_/*k*_1_ and *K*′_T_ = *k*′_2_*k*′_3_/(*k*′_1_(*k*′_2_+*k*′_3_)), we are thus assuming that the rate of the dissociation of the binding protein from the transport unit is approximately one-hundred times smaller than its translocation rate (*k*′_3_ ≈ 0.01*k*′_2_). This is a reasonable assumption because the dissociation of the binding protein from the transport unit and its movement away from the inner membrane is also limited by the slow diffusion of the binding protein [47]. Indeed, these parameter assumptions well match differences between *E*. *coli*’s maltose ABC transport system and glucose PTS (Sections C and D.3 in S1 Appendix). We thus set the translocation rate and dissociation constant to those measured for *E*. *coli*’s glucose PTS, $k_{2}^{\prime }=k_{2}=200$ sec^-1^ and $K_{T}^{\prime }=K_{T}=10$ μM. In addition, we assume that the kinetics of the binding of substate to binding protein matches that of maltose to MalE so that *K*_*D*_ = 1 μM, with association rate $k_{0f}^{\prime }=10^{5}$ mM^-1^sec^-1^ and dissociation rate $k_{0r}^{\prime }$ = 100 sec^-1^.

**Fig 4:**
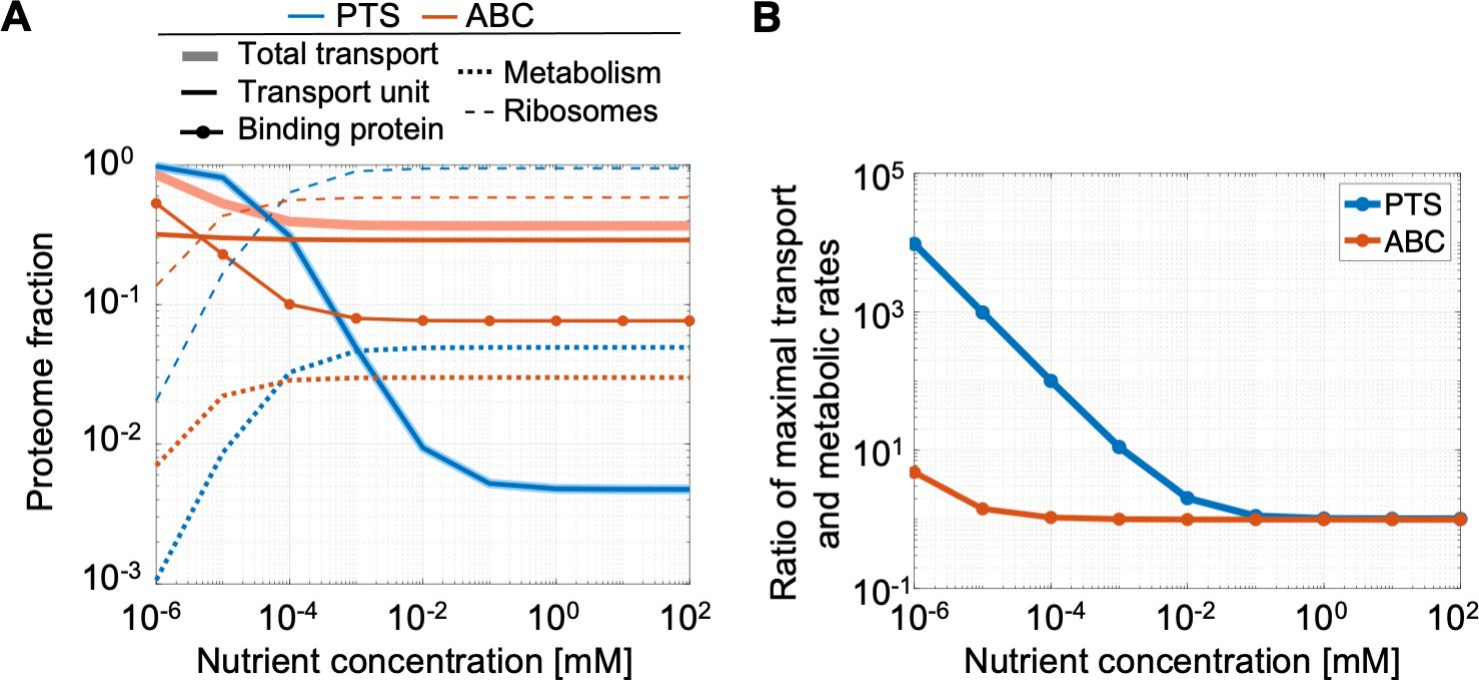
Optimal proteome allocation for PTS and ABC transport systems. Proteome fractions shown are fractions of the proteome available for the four specified protein groups. (A) While it is optimal for cells relying on either PTS or ABC transport systems to devote nearly all of their proteome to transport at low nutrient concentrations, for ABC transport systems, it is the proteome fraction of the binding proteins that increases as nutrient concentration decreases and not the fraction allocated to the membrane-bound transport units. (B) As the nutrient concentration decreases, the optimal maximal uptake of transport increases for PTS but remains constant for ABC transport systems. This results in an increasing ratio of optimal maximal uptake and maximal metabolic rates for transport by PTS as nutrient concentration decreases, while it is optimal for ABC transport systems to maintain this ratio closer to one.

**Fig 5:**
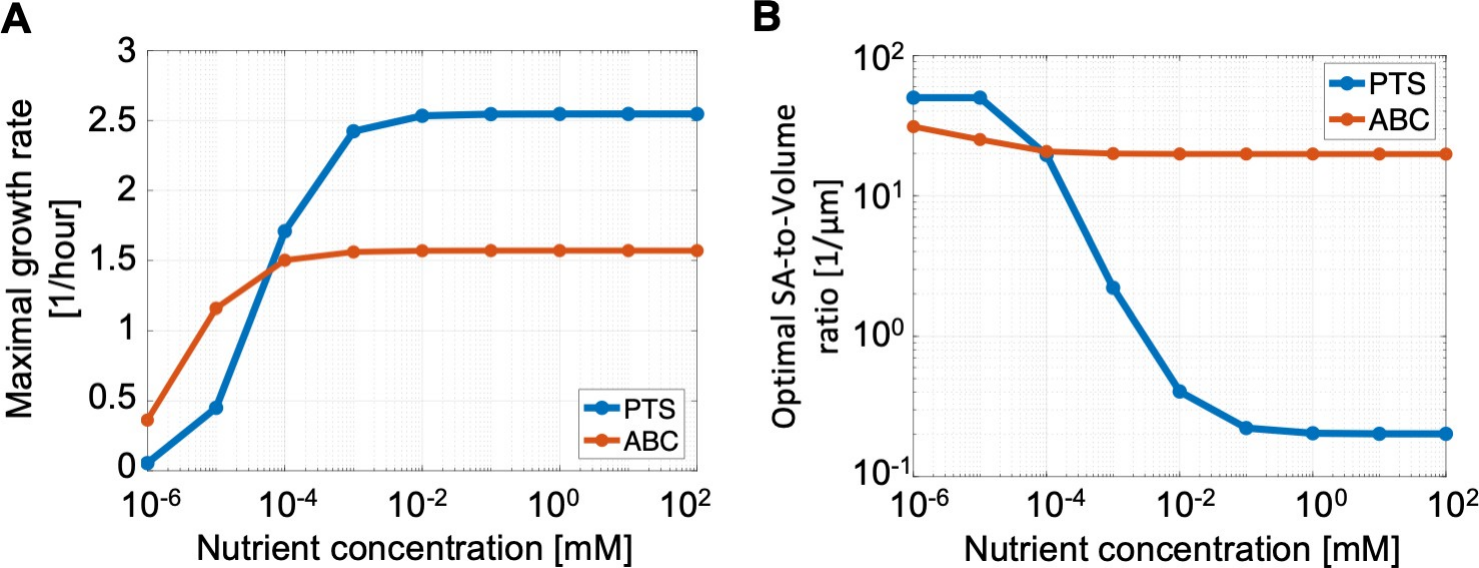
A rate–affinity trade-off. Plots show the results of proteome allocation problems using either PTS or ABC transport and solved for different extracellular nutrient concentrations (x-axis). We assume that the transport association rate is 100 times lower for ABC transport than for PTS (*k*′_1_ = 0.01*k*_1_) but that the translocation rate as well as the transport unit dissociation constant are equal ($k_{2}^{\prime }=k_{2},\;K_{T}^{\prime }=K_{T}$). We additionally limit the radius of the cell to a minimum of 60 nm, corresponding to a maximum surface-area-to-volume ratio of 50 μm^-1^. (A) shows the maximal growth rates achieved using the optimal proteome allocation, and (B) shows the optimal surface-area-to-volume ratio used to achieve those maximal growth rates. ABC transport achieves higher growth rates at low nutrient concentrations because it supports higher substrate affinities per transport proteomic cost, whereas PTS achieves higher growth rates at high nutrient concentrations because it supports higher maximal uptake rates per transport proteomic cost.

Because we assume that the diffusive rates of binding proteins limit ABC uptake rates, our model shows that PTS can achieve higher maximal uptake rates *V*_max_ per proteomic cost than ABC transport (Fig 2). Specifically, at saturating extracellular nutrient concentrations, the optimal cell using PTS devotes 80 times less proteome to transport than the optimal cell using ABC transport (Fig 4A) yet achieves a slightly (3%) higher *V*_max_ (S2 Fig). Therefore, cells using PTS achieve higher growth rates than cells using ABC transport when nutrient concentrations are high (Fig 5A).

**Fig 6:**
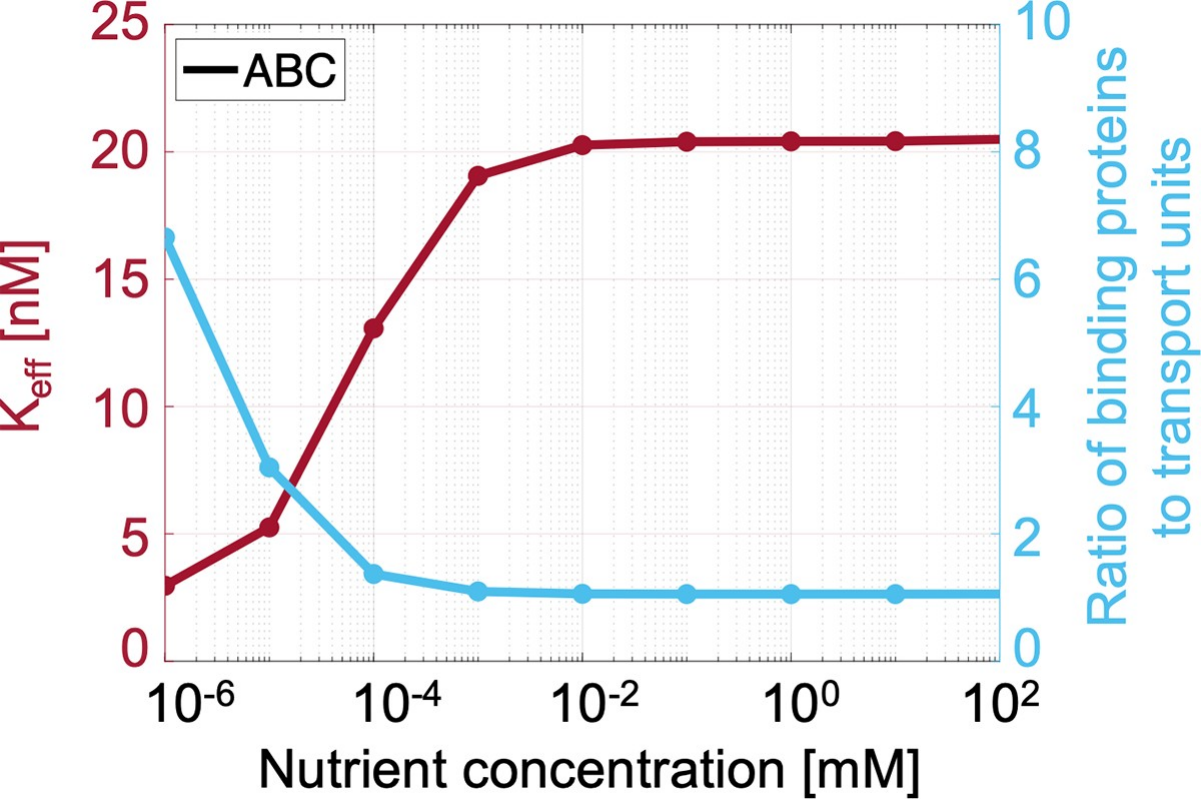
The effective half-saturation concentration of ABC transport. ABC transport systems achieve low optimal half-saturation concentrations (K_eff_, magenta curve and axis)—and thus high specific affinities—as nutrient concentrations decrease by maintaining a high surface-area-to-volume ratio and increasing the ratio of the abundance of binding proteins to the abundance of membrane-bound transport units (turquoise curve and axis). For high binding protein to transport unit ratios, the Michaelis-Menten approximation of ABC transport (Eq 6) holds (S1 Fig). At 1 nM, where the binding protein to transport unit ratio is approximately seven, the approximation gives K_eff_ ≈ 1.5 nM, while the calculated K_eff_ = 2.9 nM. (For a plot showing how we calculate the effective half-saturation concentration, see S7 Fig).

Conversely, our model shows that ABC transport systems have higher specific affinities (*a*) per proteomic cost than PTS (Figs 2 and 5A). As the nutrient concentration decreases to 1 nM, the cytoplasm of the optimal ABC cell shrinks to concentrate the limiting metabolites so that the optimal cytoplasmic concentrations remain nearly constant over all extracellular nutrient concentrations (S3 Fig). Yet the optimal periplasmic volume increases so that the optimal ABC cell at 1 nM has a periplasmic volume that is, in fact, larger than its cytoplasmic volume (S4 Fig). This increase in periplasmic volume prevents molecular overcrowding while permitting an increase in the abundance of binding proteins, which is limited by the periplasmic density constraint (Section D.1 in S1 Appendix and Figs O and P in S2 Appendix). Thus, although the optimal periplasmic binding protein concentration remains constant as nutrient levels decrease (S5 Fig), the binding protein to transport unit ratio ([BP]/[T]_total_) increases to seven (Fig 6). This increase in binding protein to transport unit ratio increases the probability that a transport unit will be bound, thus increasing uptake affinity (S6 Fig). In this way, using a binding protein with *K*_D_ = 1 *μ*M and a transport unit with dissociation constant *K*′_T_ = 10 *μ*M, the optimal ABC cell achieves an effective half-saturation concentration of *K*′_M_ ≈ 3 nM (Fig 6). Thus, although the optimal ABC cell devotes 16% less of its proteome to transport than the optimal PTS cell, it achieves a half-saturation concentration that is over three thousand times lower than the half-saturation concentration of the optimal PTS cell, *K*_M_ = *K*_T_ = 10 *μ*M. Therefore, cells using ABC transport achieve higher growth rates than cells using PTS when nutrient concentrations are low (Fig 5A).

Many bacterial species have both PTS and ABC transport systems for the same nutrient, using PTS when the nutrient is plentiful and ABC transport when the nutrient is scarce [24,49]. Because of this redundancy, it has long been hypothesized that there exists a rate-affinity trade-off between PTS and ABC transport [24,28]. Our results provide a mechanistic explanation for this trade-off and furthermore demonstrate that this trade-off, in particular, drives the differences in performance between the optimal ABC and PTS cell. Alternative hypotheses on the mechanisms creating a trade-off between the two transport mechanisms are not supported by our metabolic model. We find that the advantage of PTS in high-nutrient conditions does not stem from either lower energetic or lower proteomic costs because these costs are minimal in high-nutrient conditions. When we expanded our model to include the energetic costs of transport and furthermore incorrectly assumed that the association rate, *k*_1_, was the same for both PTS and ABC transport systems and also that the dissociation rate $k_{3}^{\prime }$ was negligible (i.e., $k_{3}^{\prime }\gg k_{2}^{\prime }$), we observed no trade-off: despite the higher proteomic and energetic costs of ABC transport, the maximal growth rate achieved by the optimal ABC cell was always greater than or equal to the maximal growth rate achieved by the optimal PTS cell (Section E in S1 Appendix).

We therefore conclude that the only trade-off that can explain the redundancy of species that utilize both PTS and ABC transport systems for the same nutrient is a rate–affinity trade-off that is a consequence of the high affinity achieved by using binding proteins and the binding proteins’ limiting rates of diffusion. Specifically, our model predicts that it is the dissociation rate of the binding protein and transport unit, $k_{3}^{\prime }$, that limits ABC uptake rate. (See sensitivity analyses in S2 Appendix.) After translocation, the bulky, unbound binding protein must dissociate from the membrane-bound transport unit and diffuse away from the inner membrane to allow a bound binding protein to associate with the transport unit. Therefore, while the translocation rate *k*_2_ governs the uptake rate by PTS, we predict that it is the diffusivity of the binding protein that governs the ABC uptake rate. The heavy reliance of heterotrophic bacteria on ABC transport systems in the oligotrophic ocean suggests that this trade-off is central to the dichotomy between the copiotrophic and oligotrophic lifestyles and that it may explain the fundamental difference in their achievable growth rates.

Our conclusion that a rate–affinity trade-off between PTS and ABC transport underpins the differentiation of oligotrophs and copiotrophs is further supported by our model’s predictions on the optimal surface-area-to-volume ratio. We find that the optimal surface-area-to-volume ratio (which is inversely proportional to the cell radius) is smaller for PTS cells in nutrient-rich conditions than it is for ABC cells in all nutrient conditions (Fig 5B). This result is in accordance with observations that typical copiotrophic marine bacteria, like *Vibrio*, are over ten times larger than typical oligotrophic ones, like SAR11 [14,40]. The model further reveals that increasing translocation rates for PTS decreases the optimal surface-area-to-volume ratio (S8 Fig). This indicates that, whereas larger surface-area-to-volume ratios allow the cell to achieve higher cytoplasmic concentrations of metabolites and proteins—and hence higher processing rates—in low-nutrient conditions, smaller surface-area-to-volume ratios are optimal at high nutrient uptake rates because they provide the cell with more space in which to process the substrate and transform it into biomass. Thus, the higher achievable uptake rates of PTS support larger optimal cell volumes.

A suite of sensitivity analyses confirmed that the fundamental trends observed from the rate-affinity trade-off depend primarily on our assumption that it is the association and dissociation rates of the binding protein and transport unit ($k_{1}^{\prime }$ and $k_{3}^{\prime }$) that limit ABC transport and not the translocation rate ($k_{2}^{\prime }$) nor the association rate of the substrate to binding protein ($k_{0f}^{\prime }$). The trends do not depend on the precise magnitude of the rates (S2 Appendix). Although our results are most sensitive to the dissociation rate of the binding protein and transport unit $k_{3}^{\prime }$, we find that a rate-affinity trade-off in the relative achievable growth rates of PTS versus ABC transport still exists when we increase the dissociation constant from $k_{3}^{\prime }$ = 0.01 $k_{2}^{\prime }$ to $k_{3}^{\prime }=k_{2}^{\prime }$ (S2 Appendix). While modifications to the surface-area-to-volume ratio and density constraints modulate the magnitude of the rate-affinity trade-off, we find that the magnitude of the trade-off is most sensitive to the relative proteomic costs of transport versus protein synthesis (S3 Appendix). If we increase the proteomic cost of transport relative to protein synthesis, then the optimal ABC cell has less of an advantage in oligotrophic conditions (that is, there is a smaller difference in the achieved growth rates of the ABC and PTS cells), whereas the optimal PTS cell has a greater advantage in high-nutrient conditions (Fig A in S3 Appendix). Conversely, if we increase the protein synthesis proteomic cost relative to the transport proteomic cost, the optimal ABC cell now has a greater advantage in oligotrophic conditions, whereas the optimal PTS cell has a smaller advantage in high-nutrient conditions (Fig B in S3 Appendix).

### ABC cells achieve high affinities while closely matching metabolic and transport capacities

Unlike a cell using PTS, a cell using ABC transport can increase its specific affinity without increasing its maximal uptake rate (Eq 6). Our model predicts that, for both the optimal PTS cell and the optimal ABC cell, as the extracellular nutrient concentration decreases, the fraction of the proteome devoted to transport increases, while the fraction of the proteome devoted to metabolic enzymes decreases (Fig 4A). For PTS, increasing the transport proteome fraction increases the maximal uptake rate *V*_max_ (Fig 2). As a result, our model demonstrates a mismatch between metabolic and transport capacities for a PTS cell optimized for growth in low-nutrient conditions: for the optimal PTS cell, the ratio of the transport capacity *V*_max_ and the metabolic capacity—which is proportional to the abundance of metabolic enzymes—exceeds one for all nutrient concentrations below 1 mM. Indeed, at 1 nM, this ratio approaches 10,000 (Fig 4B). Therefore, a PTS cell optimized for growth in nutrient-poor conditions that suddenly encounters a higher nutrient concentration would uptake more nutrient than it can process and could quickly accumulate toxic levels of metabolites if it cannot excrete them.

In contrast, as the extracellular nutrient concentration decreases, the optimal ABC cell does not allocate any additional proteome to membrane-bound transport units but only to binding proteins to increase its affinity (Fig 4A). As a result, our model shows that the optimal ABC cell maintains a transport to metabolic capacity ratio of one for all nutrient concentrations above 0.1 μM. At 1 nM, the maximum ratio is below ten (Fig 4B). Therefore, our model suggests that a cell using ABC transport is much less prone to mismatches between its proteome and the environment that may cause toxic accumulations of metabolites within the cell. Hence, cells may rarely if ever need to excrete metabolites that are consumed only using ABC transport systems.

## Discussion

We used a simple metabolic model to quantify the costs and benefits of using PTS versus ABC transport systems and thus understand the divergence of the copiotrophic and oligotrophic lifestyles of heterotrophic marine bacteria that rely on carbon as an energy source. By deriving an approximation of ABC transport in Michaelis–Menten form, we predict that, when the abundance of binding proteins sufficiently exceeds the abundance of transport units, the specific affinity of ABC transport is directly proportional not only to the transport unit abundance but also to the binding protein abundance, corroborating previous theoretical work that found that *K*_M_ is a function of binding protein abundance [28]. Our analysis thus suggests that cells should maintain high binding protein to transport unit ratios to achieve high specific affinities. We predict that, for oligotrophs such as members of the SAR11 clade, the *K*_M_ value may be over a thousand-fold smaller than the dissociation constant of the binding protein *K*_D_. Although we are aware of only two experimental studies that considered the effects of varying binding protein abundance on uptake, both provide support to our model. We used one of the experimental studies—on *E*. *coli*’s ABC maltose transport system—to directly verify our model’s predictions on the dependence of uptake on binding protein abundance (Section C in S1 Appendix). The second study found that a *Salmonella typhimurium* mutant that expresses fivefold higher levels of binding protein for histidine uptake has a fourfold lower *K*_M_—thirtyfold lower than the estimated *K*_D_ of the binding protein for histidine [50]. Our Michaelis-Menten approximation of ABC transport is consistent with these observed values, predicting that the *Salmonella* mutant’s binding protein concentration is approximately thirty times greater than the transport unit dissociation constant *K*_T_.

As ABC transport systems are ubiquitous in gram-negative bacteria, the fact that *K*_M_ may be drastically different from *K*_D_ has important implications for our ability to predict microbial dynamics. Because of the difficulty of measuring the value of *K*_M_ for uptake directly, much previous work has estimated the performance of ABC transport systems using binding assays that measure *K*_D_ instead [25,34]. Our work suggests that this estimate could differ from *K*_M_ by orders of magnitude for oligotrophs that use high abundances of binding proteins, thus potentially leading to substantial underestimates of oligotrophs’ nutrient uptake rates. In addition, a variety of microbial ecosystem models assume a constant value of *K*_M_ for uptake [51,52], but this assumption may be flawed because bacteria may vary their binding protein abundance and thus their *K*_M_ value as a function of environmental conditions. Alternatively, it is also possible that cells have evolved to express a constant binding protein abundance to maintain a constant, ecologically relevant *K*_M_ value. Experiments are needed to determine the extent to which binding protein abundance and the value of *K*_M_ vary within a species, as well as the impacts of the variability in *K*_M_ on ecosystem dynamics.

Our model provides a mechanistic explanation for the differences in performance observed between the glycine betaine transport systems of *E*. *coli* and of a SAR11 strain that is prevalent in the vast nutrient-poor expanses of the ocean [29]. The SAR11 strain can achieve nanomolar values for the half-saturation concentration of glycine betaine uptake, whereas *E*. *coli*’s genetically similar glycine betaine transport system uses a binding protein with only a micromolar dissociation constant [21]. It was posited that SAR11 achieves these higher affinities by achieving higher binding protein concentrations in a large periplasm [21]. Our model corroborates this hypothesis and furthermore demonstrates how SAR11 can achieve a nanomolar half-saturation concentration using a binding protein with the *same* micromolar binding affinity as *E*. *coli*’s glycine betaine binding protein. To achieve such a high specific affinity using a binding protein with only a micromolar dissociation constant, our model predicts that SAR11 maintains a high binding protein to transport unit ratio. Although our model cannot rule out the alternative possibility that oligotrophs evolved binding proteins with lower *K*_D_ values to achieve very low *K*_M_ values, it does demonstrate that this is not required. To determine the relative roles of low *K*_D_ values versus high binding protein abundances for achieving high affinities in SAR11, their binding proteins must be purified and used in binding assays to directly measure *K*_D_ values and contrast them with the *K*_M_ values attained from uptake rate measurements.

It was previously hypothesized that typical oligotrophs achieve higher specific affinities than copiotrophs by having higher ratios of transport units to metabolic enzymes—and thus extremely high ratios of transport to catabolic capacity [53], and this theory is still often used to explain the nutrient acquisition strategy of SAR11 [29]. Here we propose an alternative theory: unlike cells using PTS, cells using ABC transport are able to increase their specific affinity without increasing their maximal uptake rate. As a result, oligotrophs may be able to closely match their transport and metabolic capacities for a number of important compounds. We hypothesize that it is for this reason that members of the SAR11 clade experience large nutrient upshifts as toxic [9,54,55]: since transport capacity rarely exceeds metabolic capacity, they may not be able to excrete substrates consumed via ABC transport as they would not need to do so in the nutrient-poor ocean in which they evolved. Consequently, an atypical, large nutrient upshift would overwhelm the cytoplasm with substrate that the cell can neither process nor excrete.

Our metabolic model indicates that ABC transport systems are more efficient than PTS at low nutrient concentrations because expressing an additional binding protein has a lower proteomic cost than expressing an additional transport unit and, furthermore, does not incur real-estate costs on the inner membrane. Instead, the binding protein abundance is subject only to a constraint on the periplasmic density, a constraint that a cell can mitigate by modifying the fraction of its volume devoted to the periplasm. Our model predicts that the optimal periplasmic volume fraction increases as extracellular nutrient concentration decreases (S4 Fig): observations suggesting that the periplasm occupies up to 70% of the volume of a SAR11 *Pelagibacter* cell [56] are in line with this prediction. Therefore, our model predicts that a majority of an oligotroph’s proteome is comprised of binding proteins (Fig 4A). This prediction is corroborated by metaproteomic analyses showing that binding proteins are among the most prevalent bacterial proteins found in the oligotrophic ocean [19].

Our results provide a mechanistic explanation for the long-standing hypothesis of a rate–affinity trade-off for nutrient uptake by marine bacteria [57,58]. An oligotroph’s reliance on binding proteins to achieve high affinities precludes its ability to attain high growth rates because our model assumes that the rate of ABC transport is diffusion-limited due to the bulkiness of the binding proteins. In particular, our model predicts that it is the dissociation of the transport unit and binding protein that is the limiting step of ABC transport and, specifically, that this dissociation step is much slower than translocation because of the size of the binding protein. To test this hypothesis, we must measure the association and dissociation rates of binding protein and transport unit for different ABC transport systems and determine whether these rates are functions of the size of the binding protein.

We also find that the mechanism of this rate-affinity trade-off explains observations that the surface-area-to-volume ratio of a typical oligotroph, like a SAR11 cell, is at least fivefold greater than that of a typical copiotroph, like a *Vibrio* [59]. We thus propose that the high translocation rates of PTS in copiotrophs are advantageous not only because they support greater uptake rates at high nutrient concentrations but also because these higher uptake rates support larger optimal cell volumes. This is of particular importance to motile copiotrophs, which must be large enough to overcome rotational diffusion in order to swim effectively toward nutrient hotspots [60]. In addition, motile cells may not be able to attain values of *K*_M_ as low as those of oligotrophs because of the large periplasmic volume fractions that this requires. Because the distance between the outer and inner membranes dictates the length of the flagellar rotor, periplasmic volume is carefully regulated in motile cells [61] and typically does not exceed 20% of the cell volume [62].

Although this work considered optimal cell physiologies in different homogeneous, unchanging environments, it also suggests how cells may optimally regulate their proteomes and morphologies in response to changes in nutrient levels. Our analysis suggests that, in response to a decrease in nutrient concentration, a cell should shrink its cytoplasm and inflate its periplasm to increase the ratio of ABC binding proteins to transport units. It has been observed that *Vibrios*, at the onset of starvation, divide—thus shrinking in size and shedding their flagella [63,64]. It would be interesting to determine if the shedding of the flagella enables *Vibrio* to increase periplasmic volume to thus increase binding protein abundance. Similarly, although previous work suggests that SAR11 cells remodel very little of their proteome in response to environmental fluctuations [29], our model suggests that oligotrophs should regulate periplasmic volume and binding protein abundance due to the high costs of growing the outer membrane and expressing high ratios of binding proteins to transport units. Future experiments should investigate the extent to which SAR11 may vary binding protein abundance in response to nutrient levels.

In summary, our work suggests that the constraints imposed by a rate–affinity trade-off between PTS and ABC transport systems shaped the divergent evolution of copiotrophic and oligotrophic bacteria in the ocean. By quantifying this trade-off, our model helps predict the achievable nutrient uptake rates and affinities of marine heterotrophic bacteria. These mechanistic predictions could be used to constrain the parametrizations of marine microbial ecosystem models used to understand how bacterial population dynamics may affect carbon flux rates in a changing ocean.

## Methods

To compare the performance of PTS and ABC transport, we incorporated models of each (Eqs 1 and 2–5) into a single-cell metabolic model that is a modification of the self-replicator model proposed by Molenaar and others [37]. We used this model to solve the following proteome allocation problem:

$$\underset{x}{\mathrm{maximize}}\;\mu$$

subjectto:equalityconstraintsEqC.1−7,inequalityconstraintsIneq.C1–3,

$$x_{i}\geq 0\;\forall i,\;\mathrm{and}\;\mathrm{cell}\;\mathrm{radius}\;r>60\;\mathrm{nm},$$

where *μ* is the steady-state exponential growth rate; the independent variables to be optimized are *x* = (*x*_*m*_, *ϕ*, *r*, *f*_*p*_, *μ*); the vector of intracellular metabolite concentrations *x*_*m*_ = ([S]_*p*_, [S]_*c*_, [A], [W], [P]) is comprised of the periplasmic concentration of the generic carbon substrate [S]_*p*_, the cytoplasmic concentration of the carbon substrate [S]_*c*_, the cytoplasmic concentration of amino acids [A], the number of generic cell membrane units divided by the cytoplasmic volume of the cell [W], and the number of amino acids incorporated into protein divided by the cytoplasmic volume of the cell [P]; the vector *ϕ* = (*ϕ*_*BP*_, *ϕ*_*T*_, *ϕ*_*E*_, *ϕ*_*M*_, *ϕ*_*R*_) denotes the fraction of the proteome devoted to ABC binding proteins, transport units, metabolic enzymes, membrane biosynthesis enzymes, and protein synthesis enzymes respectively; and *f*_*p*_ is the fraction of the cell’s volume devoted to the periplasm.

The equality constraints 1–5 are ordinary differential equations that assume balanced, steady-state exponential growth of each of the five cellular components:

$$EqC.\;1-5:\frac{dx_{m}}{dt}=Nv_{r}-\mu x_{m}=0,$$

where *N* is a stoichiometry matrix; and *v*_*r*_ is a vector of Michaelis-Menten reaction rates,

$$v_{r}=[\begin{array}{cc} v_{diff} & \left(S_{ext}\to S_{p}\right)\\ v_{c} & \left(S_{p}\to S_{c}\right)\\ k_{E}[E]\frac{{\left[S\right]}_{c}}{K_{M,E}+{\left[S\right]}_{c}} & \left(5S_{c}\to 6A\right)\\ k_{W}[M]\frac{\left[A\right]}{K_{M,W}+[A]} & \left(A\to W\right)\\ k_{E}[R]\frac{\left[A\right]}{K_{M,R}+[A]} & \left(A\to P\right) \end{array}],$$

where enzyme concentration [*X*] = *ϕ*_*X*_*α*_*X*_[P]. We here assume that the periplasmic concentration of substrate is limited by diffusion and not by porin abundance so that the periplasmic uptake rate is

$$v_{diff}=3D\frac{{\left[S\right]}_{ext}-{\left[S\right]}_{p}}{f_{p}r^{2}},$$

where *D* is the diffusivity of the substrate and [S]_ext_ is the specified concentration of substrate in the external environment. The cytoplasmic uptake rate *v*_*c*_ is either that of PTS (Eq 1) or of ABC transport (Eqs 2–5).

Equality constraint EqC. 6 ensures that the proteome fractions sum to one:

$$EqC.\;6:1=\phi_{O,cyto}+\phi_{O,peri}+\sum_{i\in P}\phi_{i},$$

where *ϕ*_*O*,cyto_ (*ϕ*_*O*,peri_) is a required constant fraction of the proteome devoted to “other” protein components in the cytoplasm (periplasm).

Equality constraint EqC. 7 ensures that the concentration of cell membrane units, [W], is sufficient to cover both the inner and outer membranes of the cell:

$$EqC.\;7:\;4\pi \left(1+{\left(1-f_{p}\right)}^{\frac{2}{3}}\right)r^{2}=[W]\left(\frac{4\pi r^{3}}{3}\right)a_{w},$$

where *a*_*w*_ is the surface area of a single membrane unit.

Inequality constraints IneqC. 1&2 are density constraints on the cytoplasm and periplasm:

$$IneqC.\;1:\sum_{j\in M_{cyto}}m_{j}x_{m}(j)\leq \rho_{cyto},$$

$$IneqC.\;2:\sum_{j\in M_{peri}}m_{j}x_{m}(j)\leq \rho_{peri},$$

where *m*_*j*_ is the molecular weight of metabolite *j* and *ρ*_cyto_ (*ρ*_peri_) is the maximal allowed density of the cytoplasm (periplasm).

Inequality constraint IneqC. 3 ensures that the surface area of the inner membrane in sufficiently large to contain all inner membrane-bound transport units:

$${IneqC.\;3:\;f}_{SA}\left(4\pi {\left(1-f_{p}\right)}^{\frac{2}{3}}r^{2}\right)\geq [T]\left(\frac{4\pi r^{3}}{3}\right)a_{T},$$

where *f*_*SA*_ is the fraction of the surface area available for transport units and *a*_*T*_ is the surface area of a single transport unit.

Values for all of the parameters specified in this model are given and justified in Section D in S1 Appendix. To solve the optimization problem, we used MATLAB’s constrained nonlinear multivariable function solver, **fmincon**. To ensure that the solver found globally optimal solutions, we transformed the units of the constraints and variables so that their predicted magnitudes were all approximately 1 and ran the solver 50 times for each optimization problem, each time using a different initial guess for the variables *x*. The code is available at: https://github.com/noelenorris/ABC_proteome_allocation.

## Supporting information

S1 Appendix Transport and proteome allocation models. This supplemental appendix contains derivations of the PTS and ABC transport models and ABC Michaelis-Menten approximation; analysis of *E*. *coli*’s ABC maltose transport system; full exposition of metabolic model with parameter value justifications; and discussion of the energetic costs of transport. Figs A-E in the S1 Appendix support the analysis of *E*. *coli*’s ABC maltose transport system. Fig A: Effects of maltoporin abundance on uptake. Fig B: Effects of binding protein abundance on uptake. Fig C: Effects of binding protein abundance on maximal uptake rate. Fig D: Effects of binding protein abundance on the half-saturation concentration of uptake. Fig E: The permeability of the outer membrane limits half-saturation concentration of uptake.
(PDF)

S2 Appendix Sensitivity analyses of ABC transport system. This supplemental appendix presents Figs A-V, showing the optimal solutions of the ABC cell when specified parameter values are modified against the baseline value. Fig A: Sensitivity analysis, *k*′_2_. Fig B: Sensitivity analysis, *k*′_2_: optimal proteome fractions. Fig C: Sensitivity analysis, *k*′_1_. Fig D: Sensitivity analysis, *k*′_1_: optimal proteome fractions. Fig E: Sensitivity analysis, *k*′_3_. Fig F: Sensitivity analysis, *k*′_3_: optimal proteome fractions. Fig G: Sensitivity analysis, *k*′_1_ and *k*′_3_. Fig H: Sensitivity analysis, *k*′_1_ and *k*′_3_: optimal proteome fractions. Fig I: Sensitivity analysis, *k*′_0*f*_. Fig J: Sensitivity analysis, *k*′_0*f*_: optimal proteome fractions. Fig K: Sensitivity analysis, ∅_*O*,*cyto*_. Fig L: Sensitivity analysis, ∅_*O*,*cyto*_: optimal proteome fractions. Fig M: Sensitivity analysis, *ρ*_*cyto*_. Fig N: Sensitivity analysis, *ρ*_*cyto*_: optimal proteome fractions. Fig O: Sensitivity analysis, *ρ*_*peri*_. Fig P: Sensitivity analysis, *ρ*_*peri*_: optimal proteome fractions. Fig Q: Sensitivity analysis, *f*_*SA*_. Fig R: Sensitivity analysis, *f*_*SA*_: optimal proteome fractions. Fig S: Sensitivity analysis, number of amino acids comprising binding protein. Fig T: Sensitivity analysis, number of amino acids comprising binding protein: optimal proteome fractions. Fig U: Sensitivity analysis, *D*. Fig V: Sensitivity analysis, *D*: optimal proteome fractions.
(PDF)

S3 Appendix Sensitivity analyses of rate-affinity trade-off. This supplemental appendix presents Figs A-G, which assesses the sensitivity of the rate-affinity trade-off by contrasting the optimal ABC and PTS cells when particular parameters are modified. Fig A: PTS versus ABC, transport proteomic costs x10. Fig B: PTS versus ABC, protein synthesis proteomic cost x10. Fig C: PTS versus ABC, ∅_*O*,*cyto*_ = 0.5. Fig D: PTS versus ABC, *ρ*_*cyto*_ x0.01. Fig E: PTS versus ABC, *ρ*_*cyto*_ x100. Fig F: PTS versus ABC, *ρ*_*peri*_ x0.01. Fig G: PTS versus ABC, *ρ*_*peri*_ x100
(PDF)

S1 Fig Comparison of approximate and exact ABC transport half-saturation concentration values. Here we compare our Michaelis-Menten approximation of the half-saturation concentration for ABC transport with the exact half-saturation concentration obtained by solving the set of four equations for ABC transport rates using baseline values for the kinetics rates and modifying the periplasmic concentration of transport units and binding proteins. Note that, for a periplasmic transport unit concentration of 1.16 mM, the half-saturation concentration does not asymptote to the approximation because, in this case, [T]_total_>*k*′_0*r*_/*k*′_1*f*_. Yet the exact solution follows the same trend as the approximation.
(TIF)

S2 Fig Optimal maximal uptake rates and specific affinities. Here are plots showing the optimal maximal uptake rates, *V*_*max*_, and corresponding optimal specific affinities, *V*_*max*_/*K*_*M*_.
(TIF)

S3 Fig Optimal cytoplasmic concentrations for ABC cell. Optimal cytoplasmic concentrations of intracellular nutrient (S_c_), amino acids, and total protein (in units of amino acids) over extracellular nutrient condition for cell with ABC transport. Although the extracellular nutrient concentration varies over many magnitudes, the optimal intracellular concentrations vary by less than a factor of three.
(TIF)

S4 Fig Optimal periplasmic volume fraction for ABC transport. The optimal periplasmic volume fraction increases as nutrient concentration decreases to allow for greater abundances of binding proteins, which are subject to a density constraint on the periplasm.
(TIF)

S5 Fig Optimal periplasmic concentrations for ABC cell. Although the optimal binding protein concentration remains nearly constant over all extracellular nutrient concentrations, the periplasmic transport unit concentration ([T]_total_) decreases as nutrient concentration decreases due to the inflation of the periplasm. While the periplasm inflates, the cytoplasm shrinks so that, for an extracellular nutrient concentration of 1 nM, the optimal periplasmic concentration of transport units is less than the abundance of transport units divided by the cytoplasmic volume ([T]_total_V_peri_/V_cyto_).
(TIF)

S6 Fig Impact of modifications to periplasmic volume around optimal solution of ABC cell at nutrient concentration of 1 nM. To understand why the periplasm inflates as the nutrient concentration decreases to 1 nM, we plot the proportion of bound transport units (A) and effective half-saturation constant, K_M_, (B) as we modify the periplasmic volume of the optimal solution for a nutrient concentration of 1 nM. We assumed that both the concentration of binding proteins in the periplasm and the abundance of transport units on the inner membrane remain constant. Therefore, as the periplasm grows, the periplasmic concentration of transport units decreases and the ratio of binding proteins to transport units increases. (A) shows how the increase in abundance of binding proteins due to the inflation of the periplasm leads to an increase in the proportion of bound transport units, where we here assume that the concentration of free substrate in the periplasm ([S]_p_) is equal to 1 nM. (B) shows the calculated half-saturation constant by fitting the Michaelis-Menten equation to the exact solutions of ABC transport uptake (Eqs 2 to 5), as well as our Michaelis-Menten approximation of the half-saturation constant (Eq 6), which holds only when the binding protein concentration sufficiently exceeds the transport unit concentration.
(TIF)

S7 Fig Calculating the half-saturation concentration of ABC transport. To calculate the effective half-saturation concentration of an optimal solution to a particular proteome allocation problem, we used the system of equations describing ABC transport to determine the uptake rate over a range of nutrient concentrations (*x*-axis). Here we show the calculated uptake rates over various nutrient concentrations for the proteome allocation obtained when optimized the cell for growth at an extracellular concentration of [*S*]_*ext*_ = 1 nM.
(TIF)

S8 Fig Sensitivity analysis of proteome allocation for PTS transport to changes in translocation rate *k*_2_ at extracellular carbon concentration [S]_ext_ = 100 mM. Increases in the translocation rate result in (A) higher achievable growth rates and (B) larger optimal cell radii (that is, smaller surface-area-to-volume ratios).
(TIF)

S9 Fig Active constraints on cell radius. Both the surface area “real estate" constraints and the density constraints are active for the PTS transport proteome allocation problem. Increases in maximal allowed density result in smaller optimal cell radii (red and yellow). Increases in the fraction of the surface area available to the membrane-bound transport units result in larger optimal cell radii (purple and green).
(TIF)
