# Supplementary material for: Mechanistic model of nutrient uptake explains dichotomy between marine oligotrophic and copiotrophic bacteria
Source: PLoS Comput Biol. 2021 May 19;17(5):e1009023. doi: 10.1371/journal.pcbi.1009023 (PMC8168909; doi:10.1371/journal.pcbi.1009023)
Supplement: S2 Appendix — This supplemental appendix presents Figs A-V, showing the optimal solutions of the ABC cell when specified parameter values are modified against the baseline value. Fig A: Sensitivity analysis, k′2. Fig B: Sensitivity analysis, k′2: optimal proteome fractions. Fig C: Sensitivity analysis, k′1. Fig D: Sensitivity analysis, k′1: optimal proteome fractions. Fig E: Sensitivity analysis, k′3. Fig F: Sensitivity analysis, k′3: optimal proteome fractions. Fig G: Sensitivity analysis, k′1 and k′3. Fig H: Sensitivity analysis, k′1 and k′3: optimal proteome fractions. Fig I: Sensitivity analysis, k′0f. Fig J: Sensitivity analysis, k′0f: optimal proteome fractions. Fig K: Sensitivity analysis, ∅O,cyto. Fig L: Sensitivity analysis, ∅O,cyto: optimal proteome fractions. Fig M: Sensitivity analysis, ρcyto. Fig N: Sensitivity analysis, ρcyto: optimal proteome fractions. Fig O: Sensitivity analysis, ρperi. Fig P: Sensitivity analysis, ρperi: optimal proteome fractions. Fig Q: Sensitivity analysis, fSA. Fig R: Sensitivity analysis, fSA: optimal proteome fractions. Fig S: Sensitivity analysis, number of amino acids comprising binding protein. Fig T: Sensitivity analysis, number of amino acids comprising binding protein: optimal proteome fractions. Fig U: Sensitivity analysis, D. Fig V: Sensitivity analysis, D: optimal proteome fractions. (PDF) [file pcbi.1009023.s002.pdf]

Mechanistic model of nutrient uptake explains dichotomy between marine oligotrophic and copiotrophic bacteria

S2 Appendix: Sensitivity analyses of ABC transport system

Noele Norris, Naomi M. Levine, Vicente I. Fernandez, Roman Stocker

Code is accessible at: `https://github.com/noelenorris/ABC_proteome_allocation`

Contents

A ABC kinetics **1**
A.1 Translocation rate, k_2' 1
A.2 Transporter association rate, k_1' 4
A.3 Transporter dissociation rate, k_3' 6
A.4 Transporter association and dissociation rates, k_1' and k_3' 8
A.5 Binding protein association rate, k_{0f} 10

B Cell constraints **12**
B.1 Fraction of proteome devoted to other cytoplasmic proteins, $\phi_{\mathrm{O,cyto}}$ 12
B.2 Cytoplasmic density, ρ_{cyto} 14
B.3 Periplasmic density, ρ_{peri} 16
B.4 Available surface area, f_{SA} 18

C Binding protein proteomic cost **20**

D Substrate diffusivity **22**

In the sections below, we present the optimal solutions of the ABC cell when the specified parameter value is modified against the baseline value. We show the baseline PTS maximal growth rates for reference.

A ABC kinetics

A.1 Translocation rate, k_2'

We find that increasing the translocation rate k_2' does not improve performance because we assume that the limiting steps of ABC transport are the association and dissociation of the binding protein and transport unit.

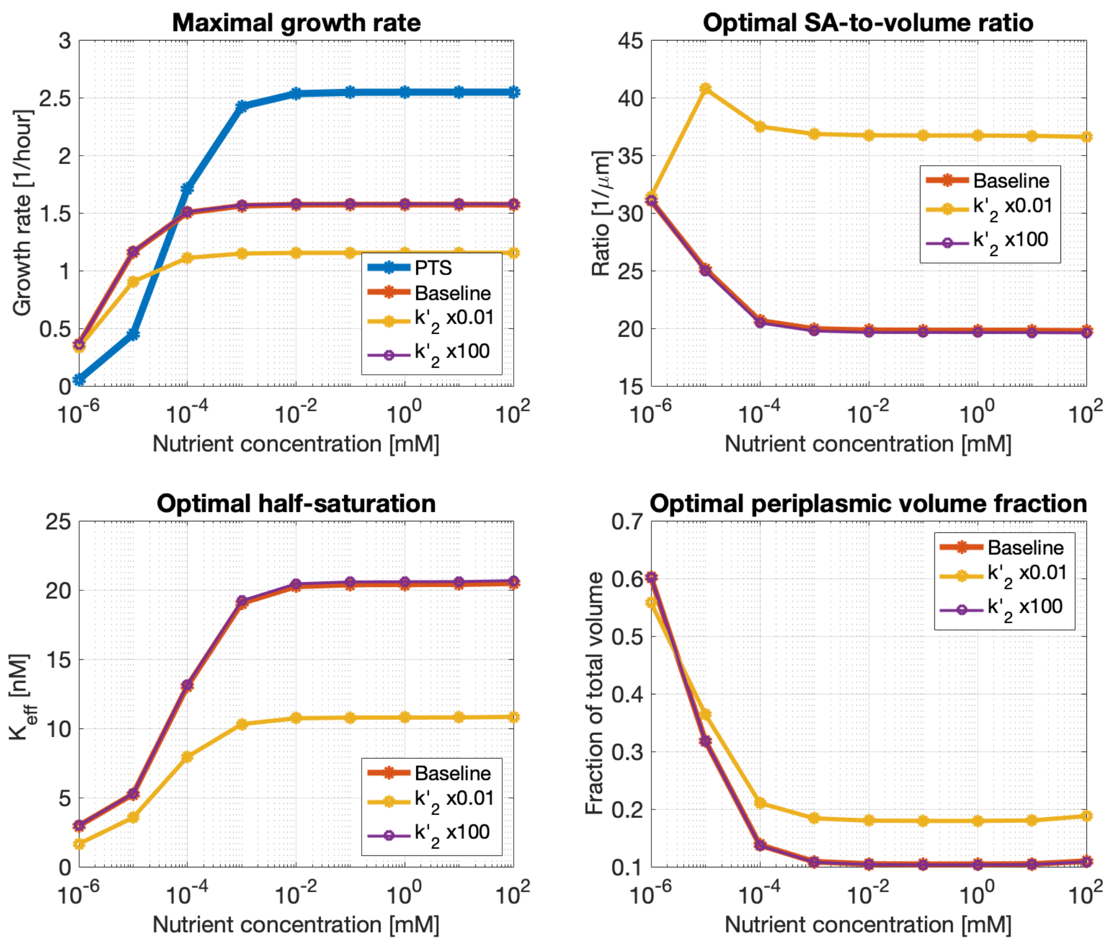

Fig A. Sensitivity analysis, k_2':

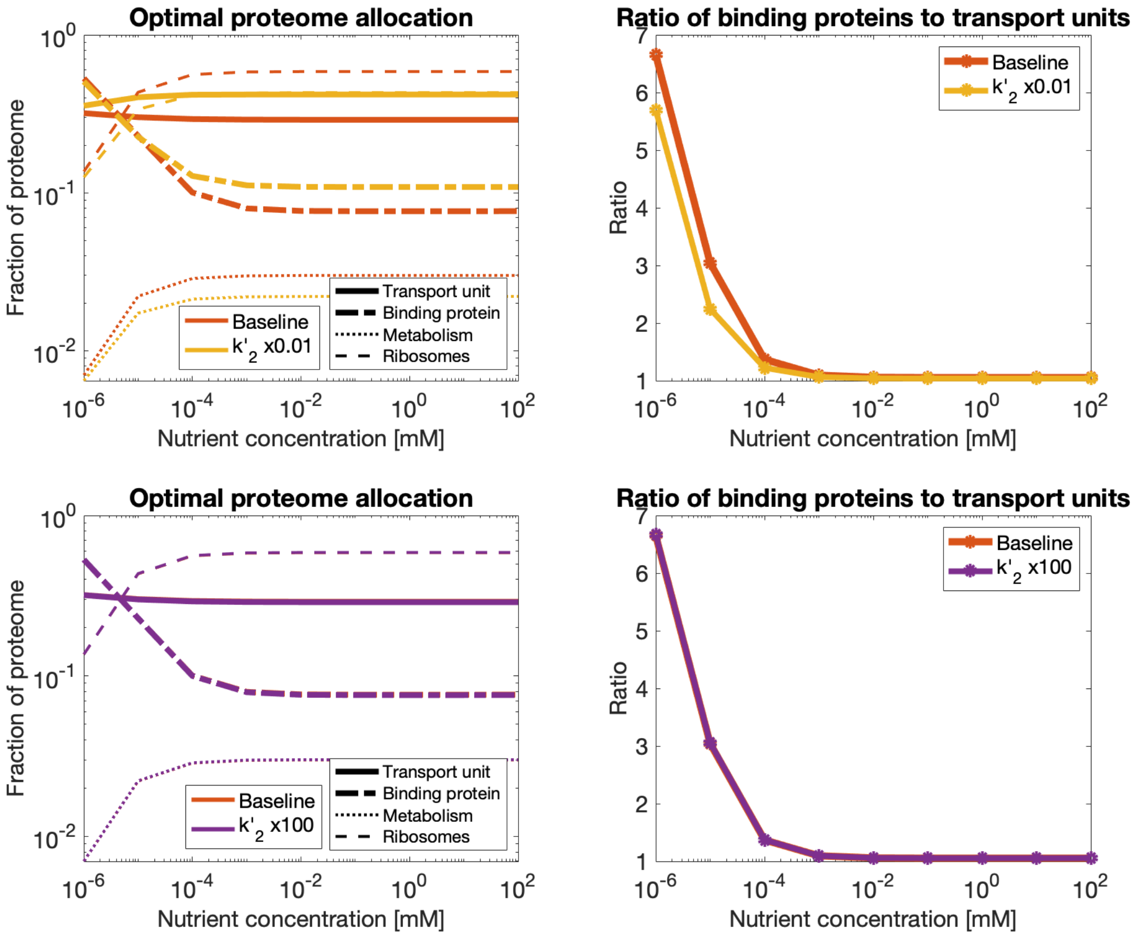

Fig B. Sensitivity analysis, k_2': optimal proteome fractions.

A.2 Transporter association rate, k'_1

Note that, for $k'_1 \times 10$, $K'_T \times 0.1$, and, for $k'_1 \times 100$, $K'_T \times 0.01$.

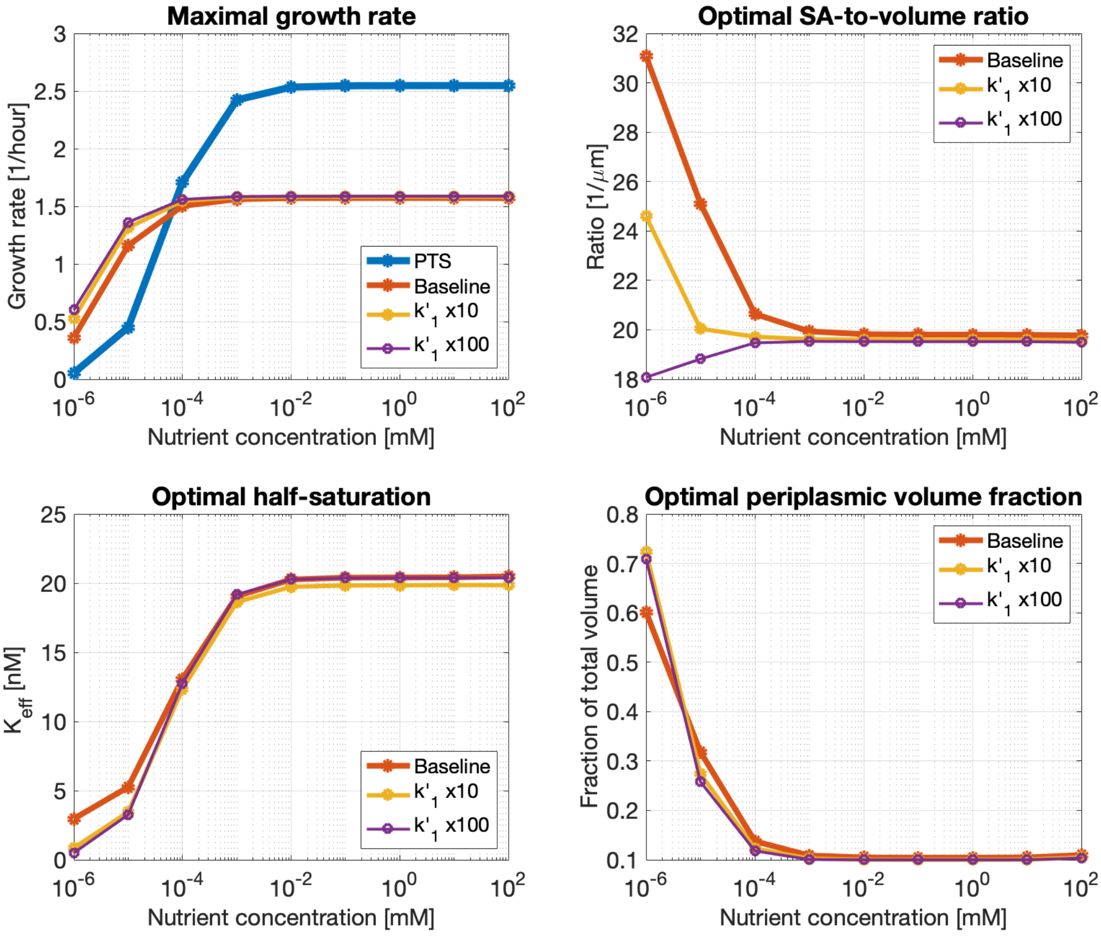

Fig C. Sensitivity analysis, k'_1

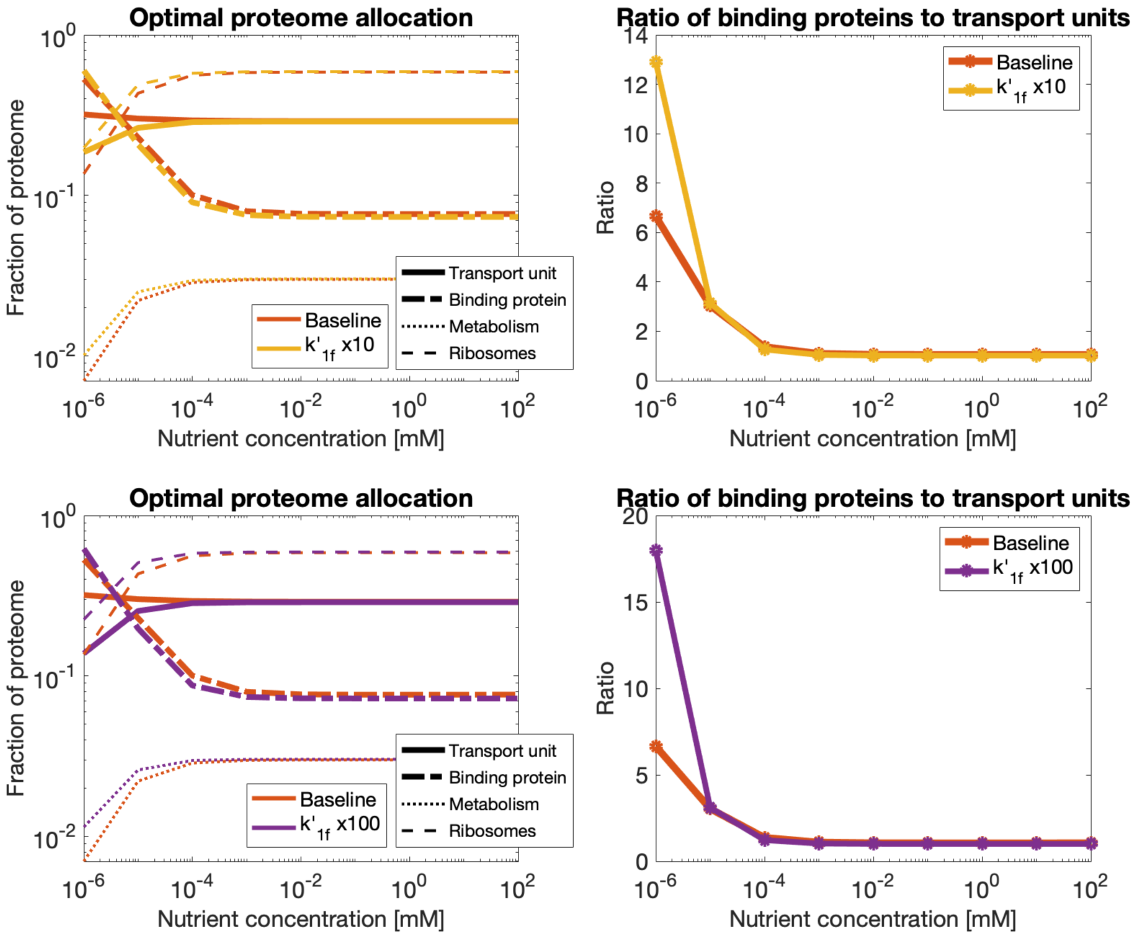

Fig D. Sensitivity analysis, k_1': optimal proteome fractions.

A.3 Transporter dissociation rate, k_3'

Among the transport kinetics parameters, our results are most sensitive to the dissociation rate k_3'. Because we assume that k_3' cannot be greater than the translocation rate k_2', the existence of this additional reaction step still limits the maximal growth rate relative to PTS. In addition, because increasing the rate increases the dissociation constant K_T', the optimal half-saturation constant is sensitive to k_3'.

Note that, for $k_3' \times 10$, $K_T' \times 9.2$, and, for $k_3' \times 100$, $K_T' \times 50.5$.

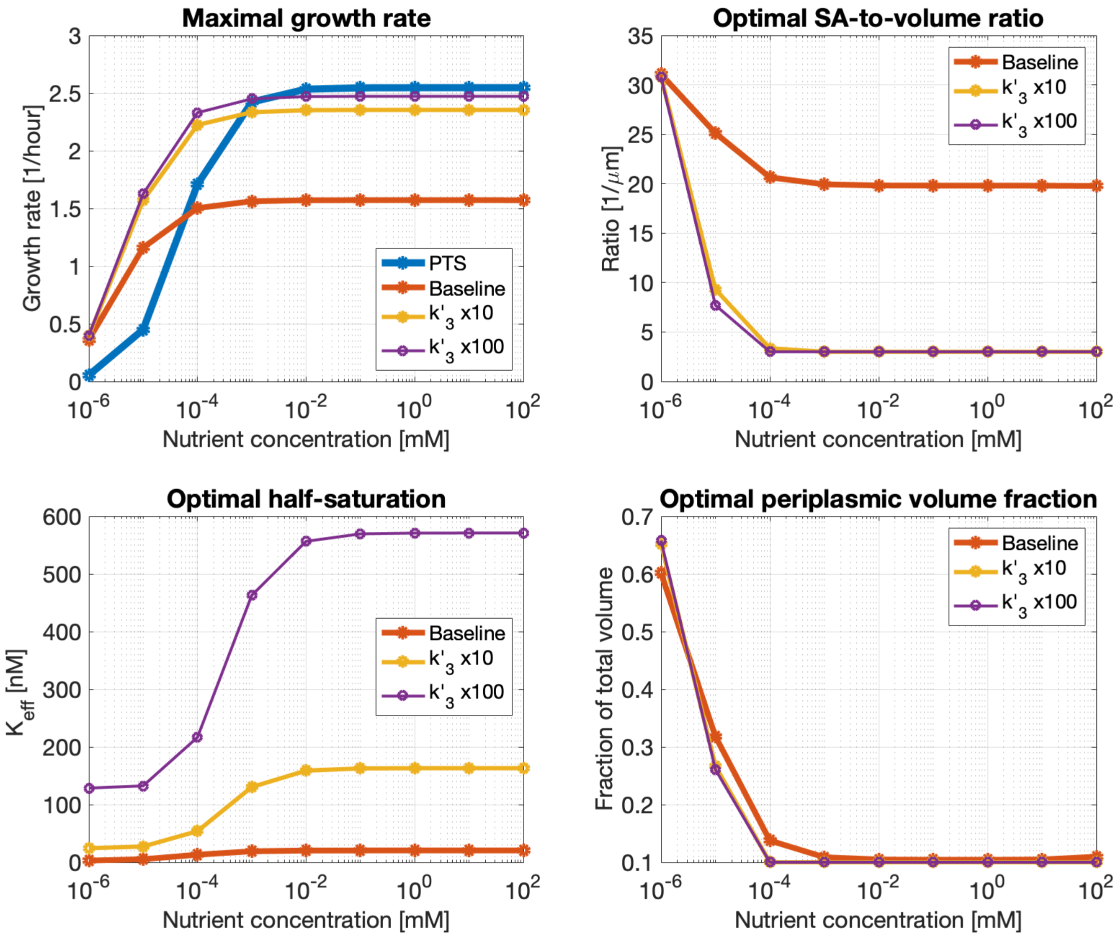

Fig E. Sensitivity analysis, k_3'

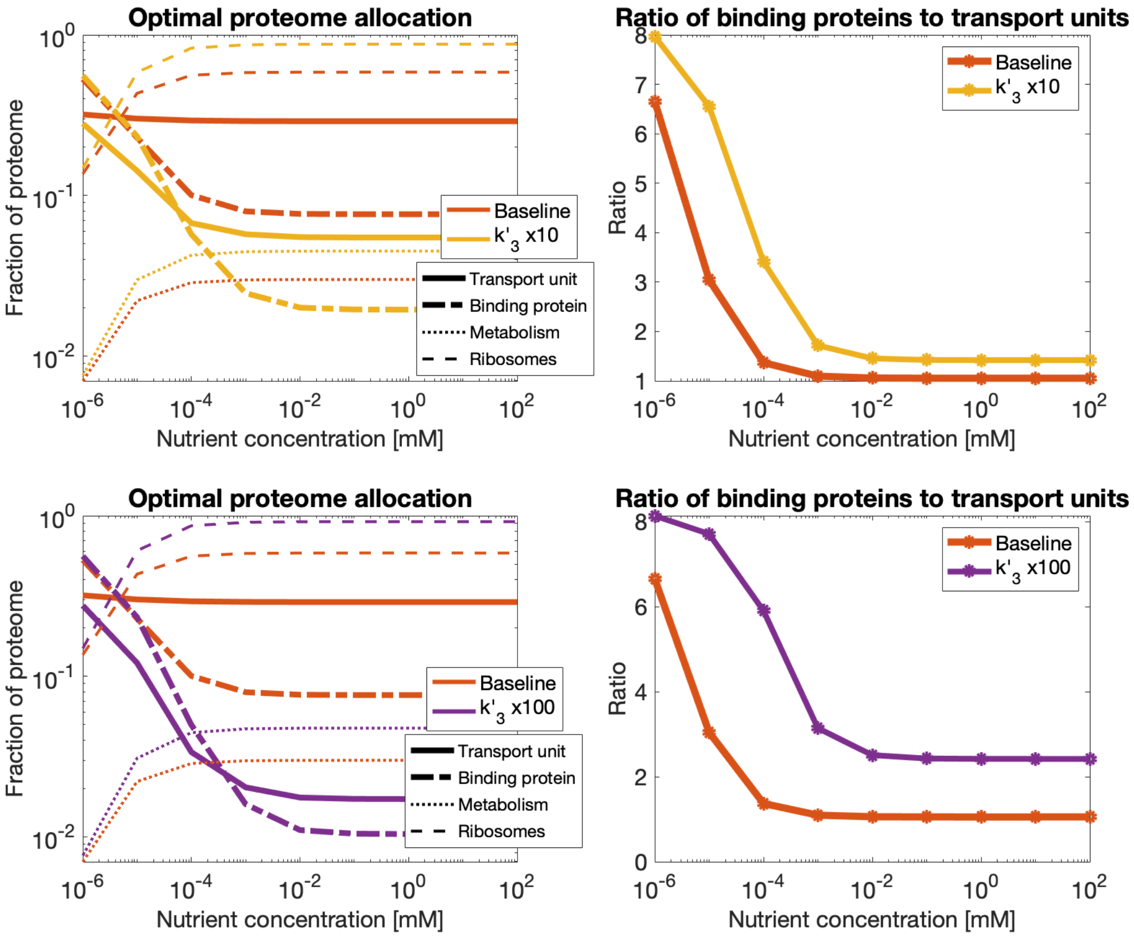

Fig F. Sensitivity analysis, k_3': optimal proteome fractions.

A.4 Transporter association and dissociation rates, k_1' and k_3'

Here we modify k_1' and choose k_3' so that the transport dissociation constant remains the same, $K_T' = K_T$. Note that, for increasing values of k_1' and k_3', the model predicts smaller optimal surface area to volume ratios and much higher ratios of binding proteins to transport units.

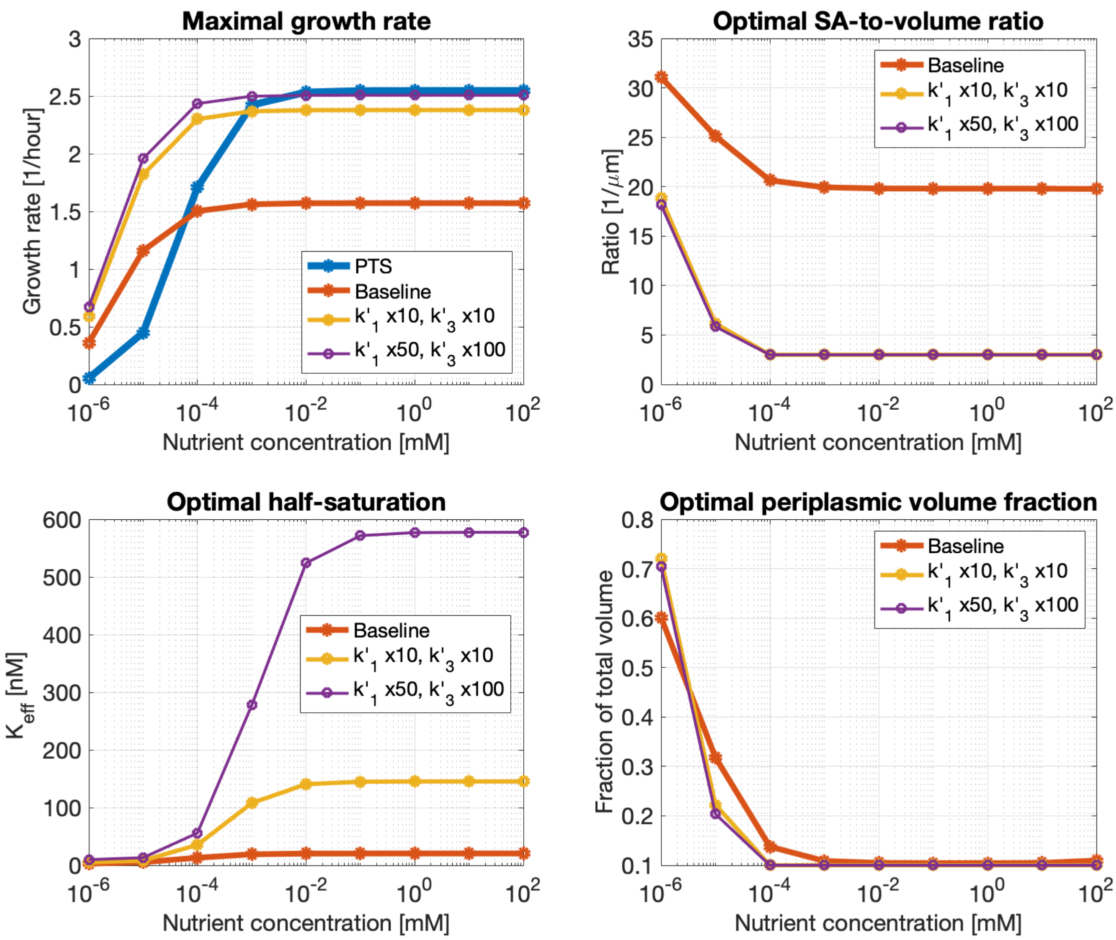

Fig G. Sensitivity analysis, k_1' and k_3'

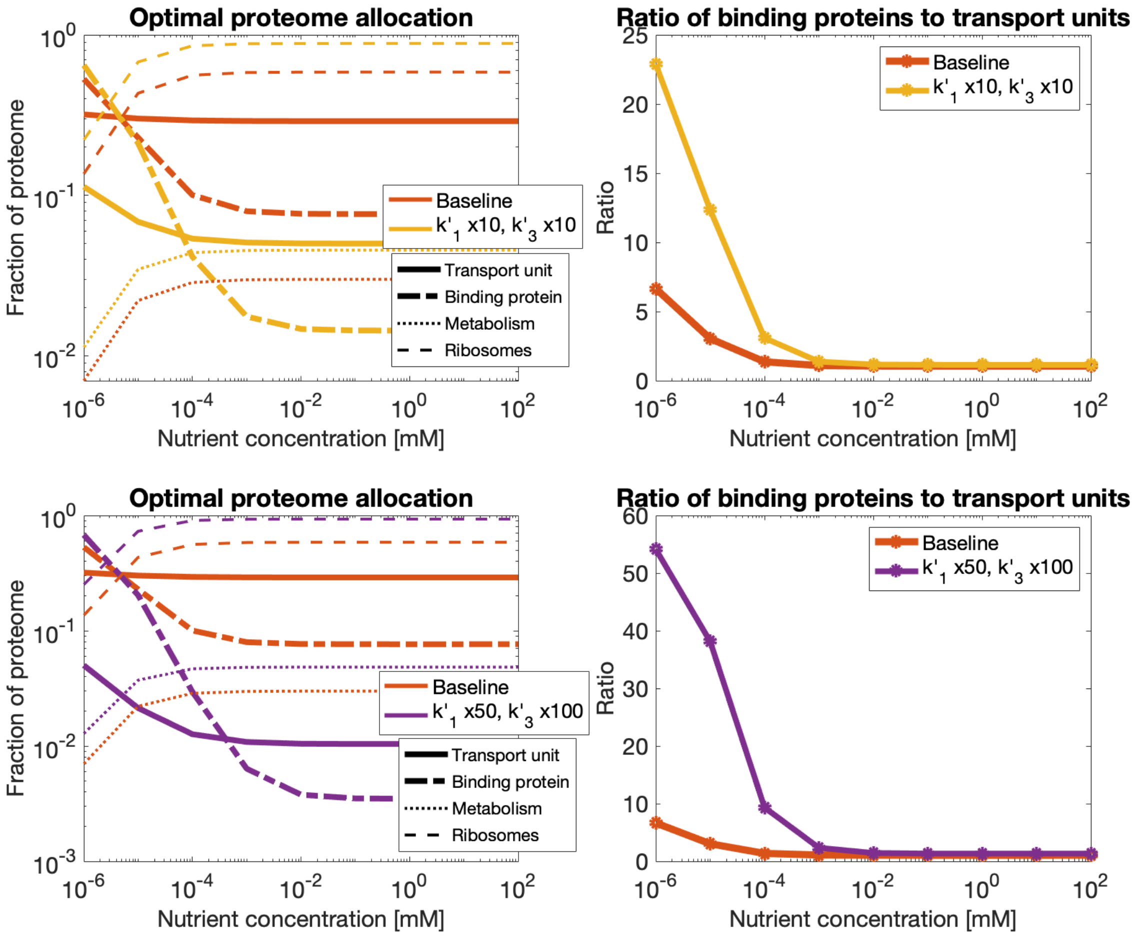

Fig H. Sensitivity analysis, k_1' and k_3': optimal proteome fractions.

A.5 Binding protein association rate, k_{0f}

Note that for $k_{0\mathrm{f}} \times 0.1$, $K_{\mathrm{D}} = 10\ \mu\mathrm{M}$, and, for $k_{0\mathrm{f}} \times 10$, $K_{\mathrm{D}} = 0.1\ \mu\mathrm{M}$.

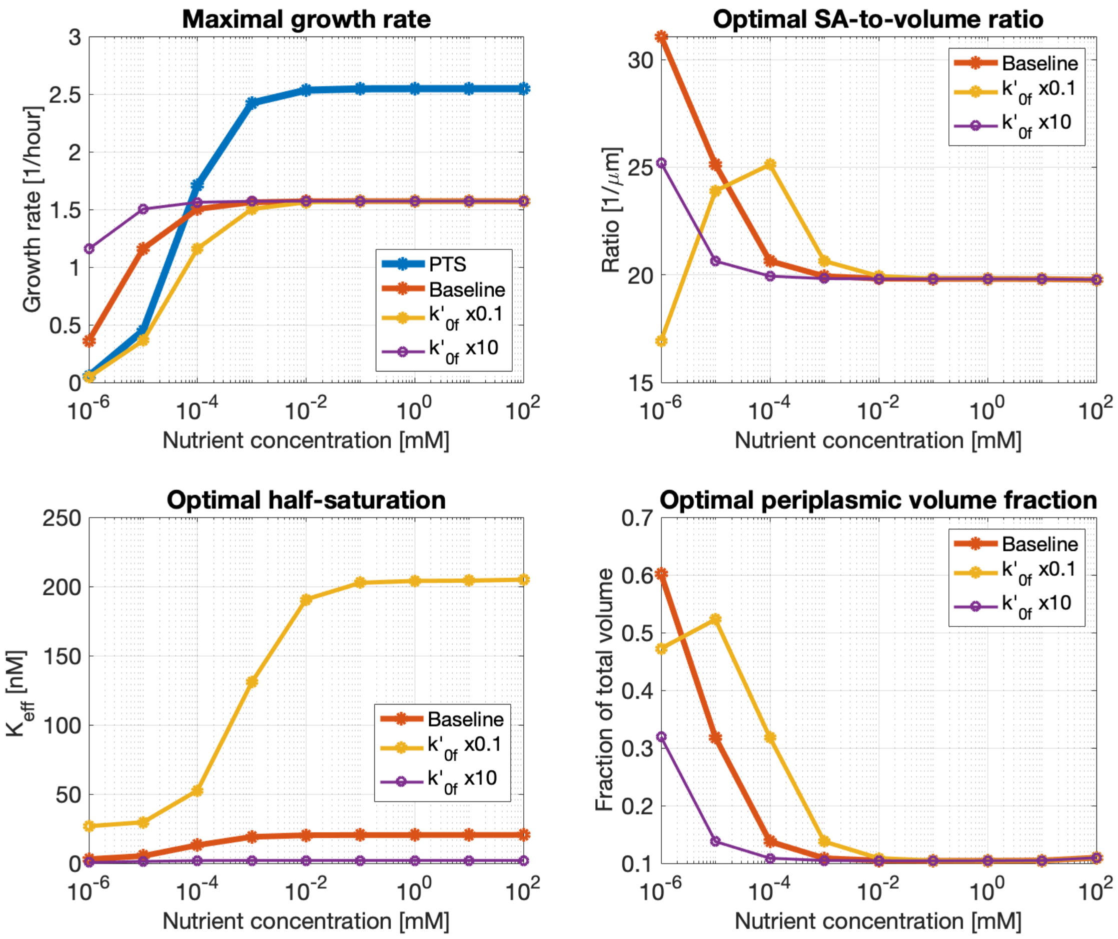

Fig I. Sensitivity analysis, k'_{0f}:

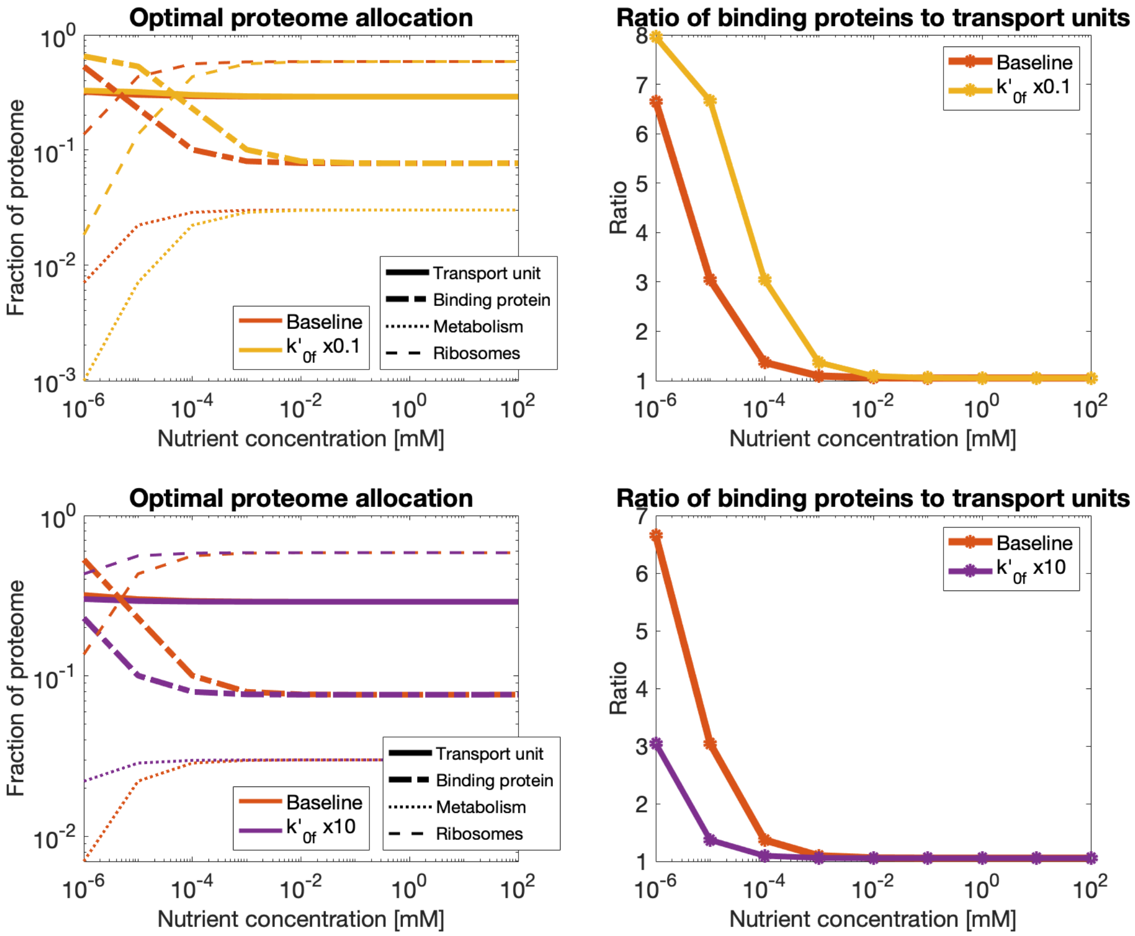

Fig J. Sensitivity analysis, k'_{0f}: optimal proteome fractions.

B Cell constraints

B.1 Fraction of proteome devoted to other cytoplasmic proteins, $\phi_{O,cyto}$

As $\phi_{O,cyto}$ increases, the optimal SA-to-volume ratio decreases, scaling with growth rate, as the cell requires more and more of its proteome fraction in the cytoplasm.

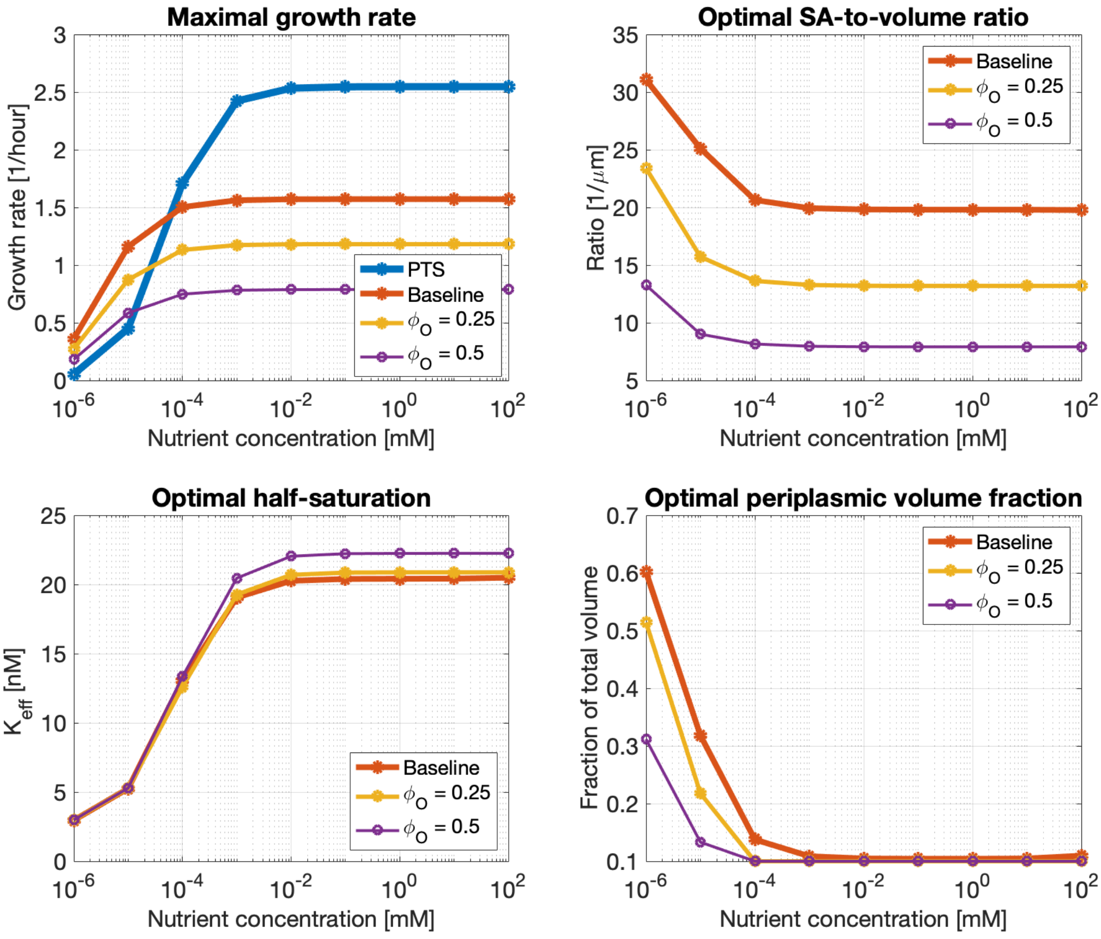

Fig K. Sensitivity analysis, $\phi_{O,cyto}$

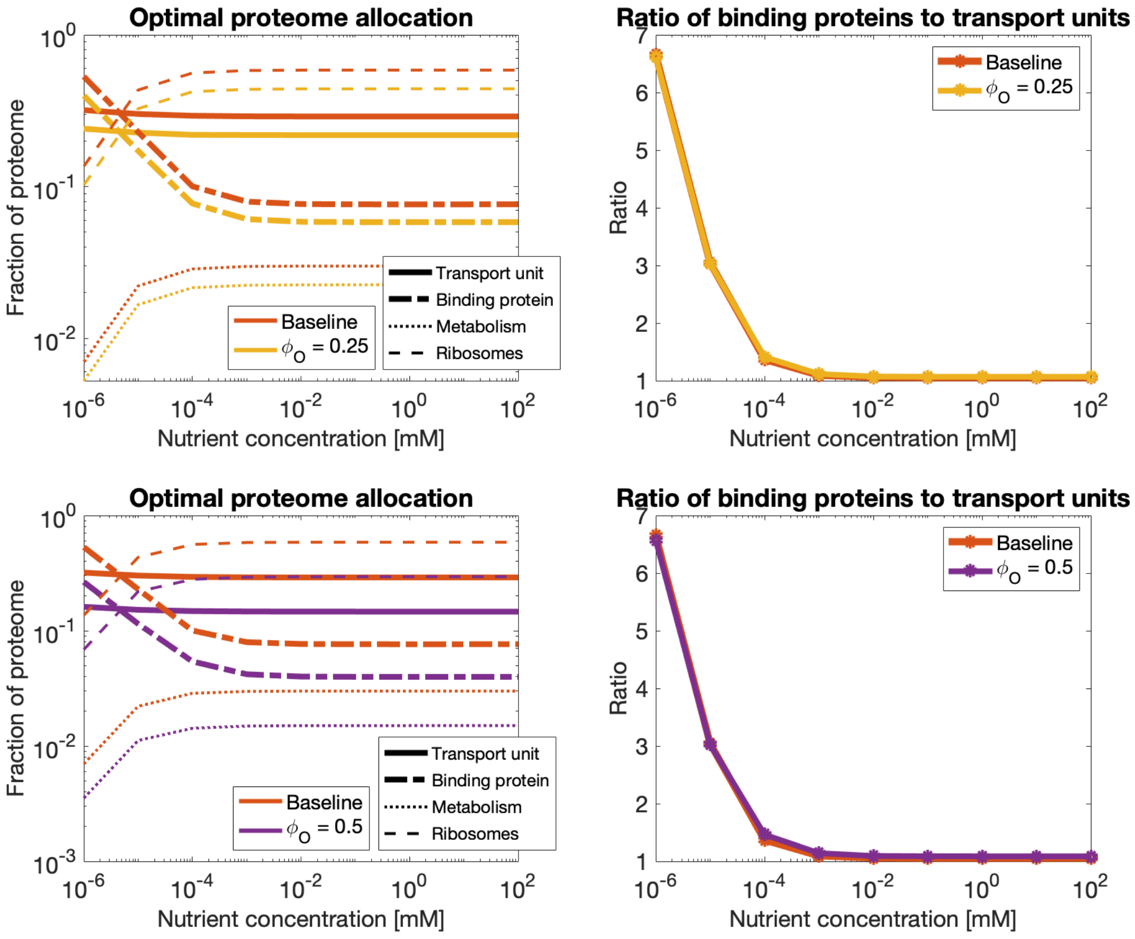

Fig L. Sensitivity analysis, $\phi_{\mathbf{O,cyto}}$: optimal proteome fractions.

B.2 Cytoplasmic density, ρ_{cyto}

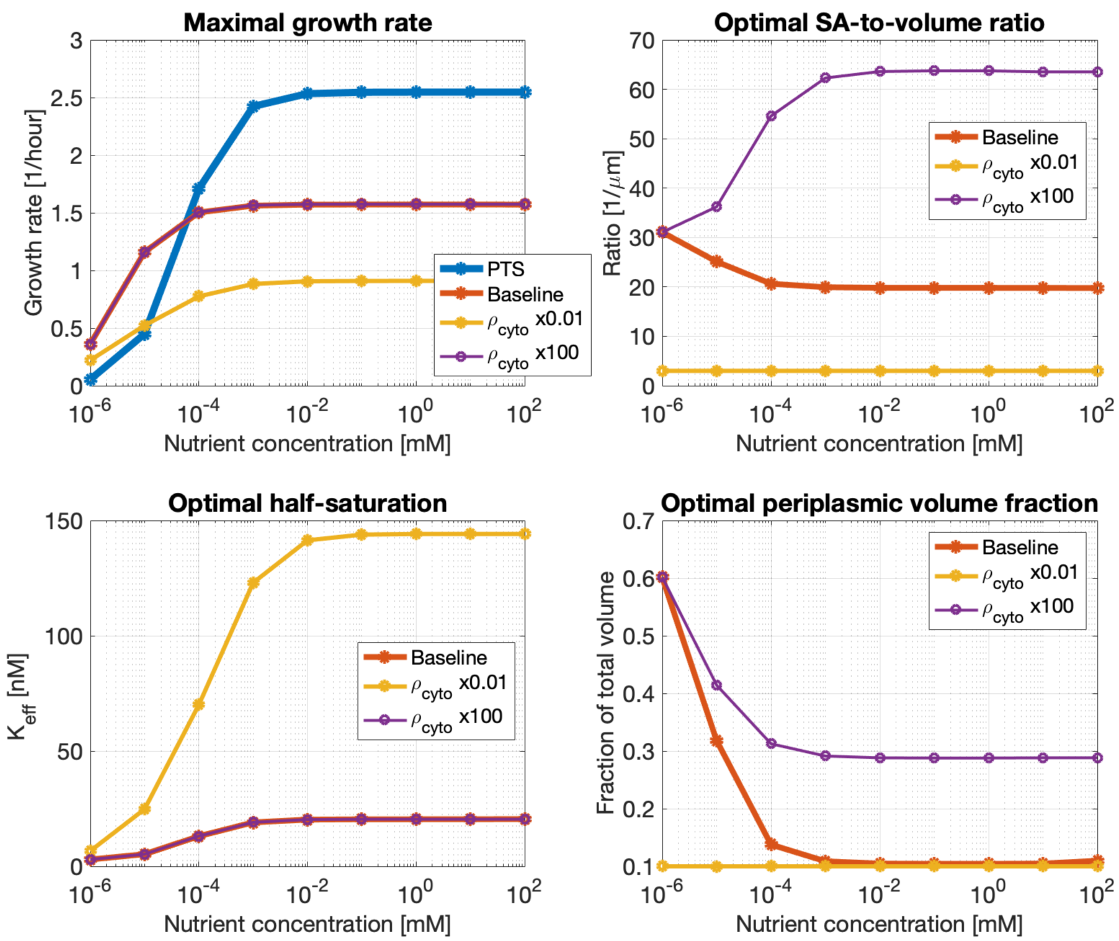

Fig M. Sensitivity analysis, ρ_{cyto}

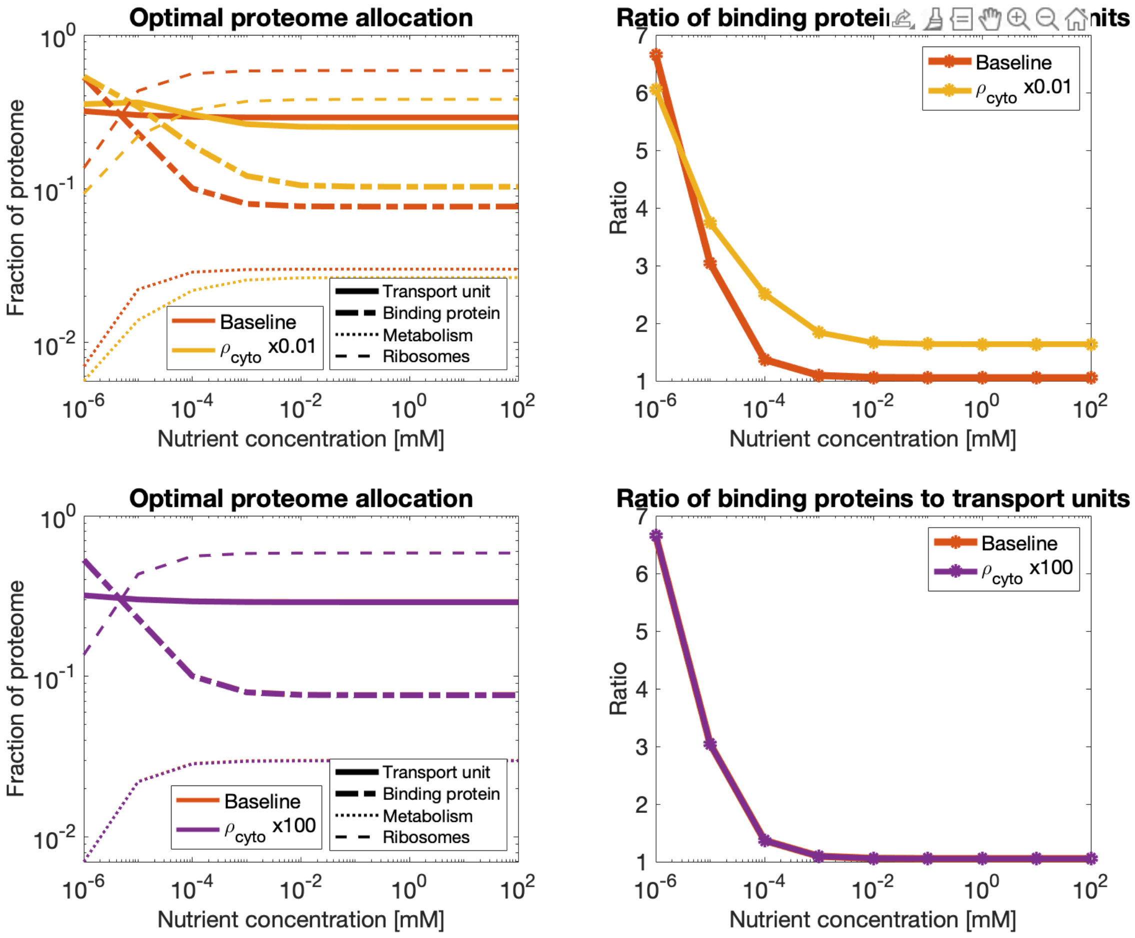

Fig N. Sensitivity analysis, $\rho_{\mathbf{cyto}}$: optimal proteome fractions.

B.3 Periplasmic density, ρ_{peri}

The optimal periplasmic volume fraction strongly depends on the maximal periplasmic density, ρ_{peri}.

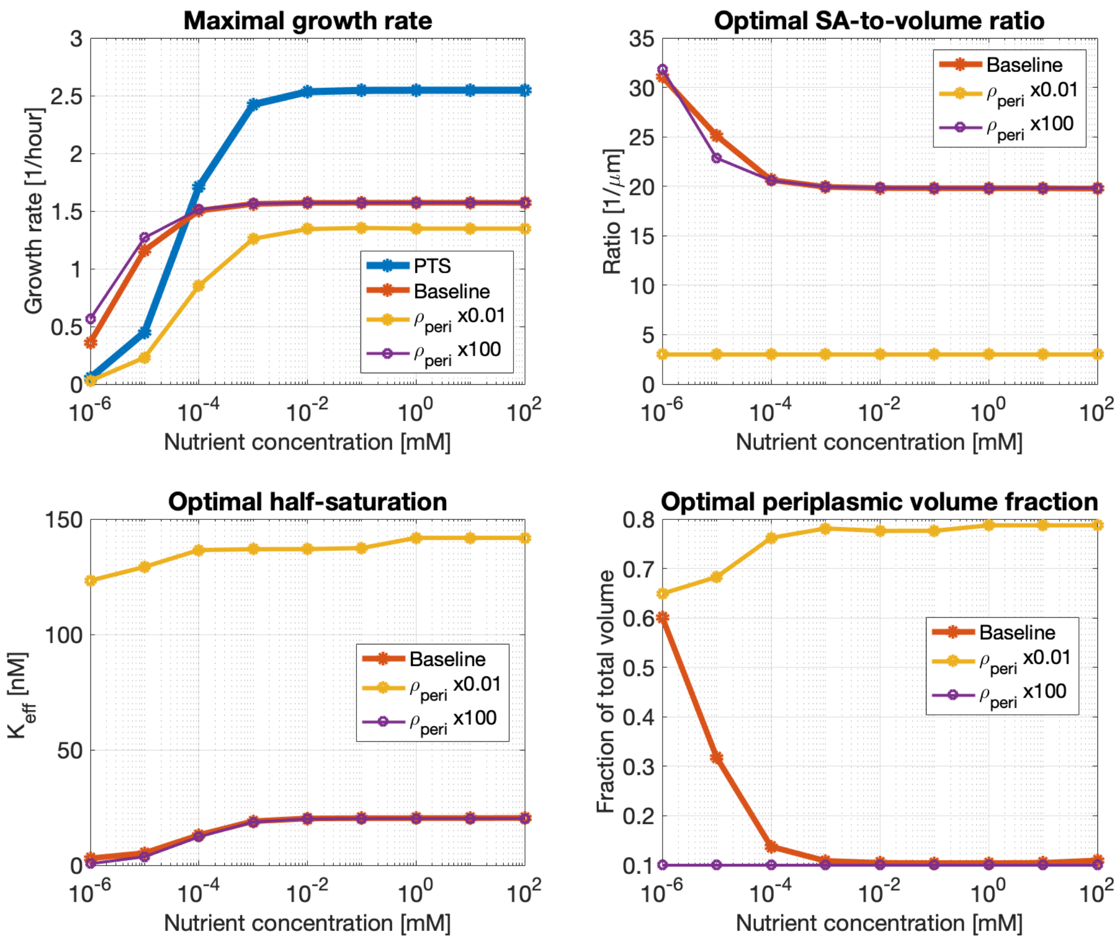

Fig O. Sensitivity analysis, ρ_{peri}

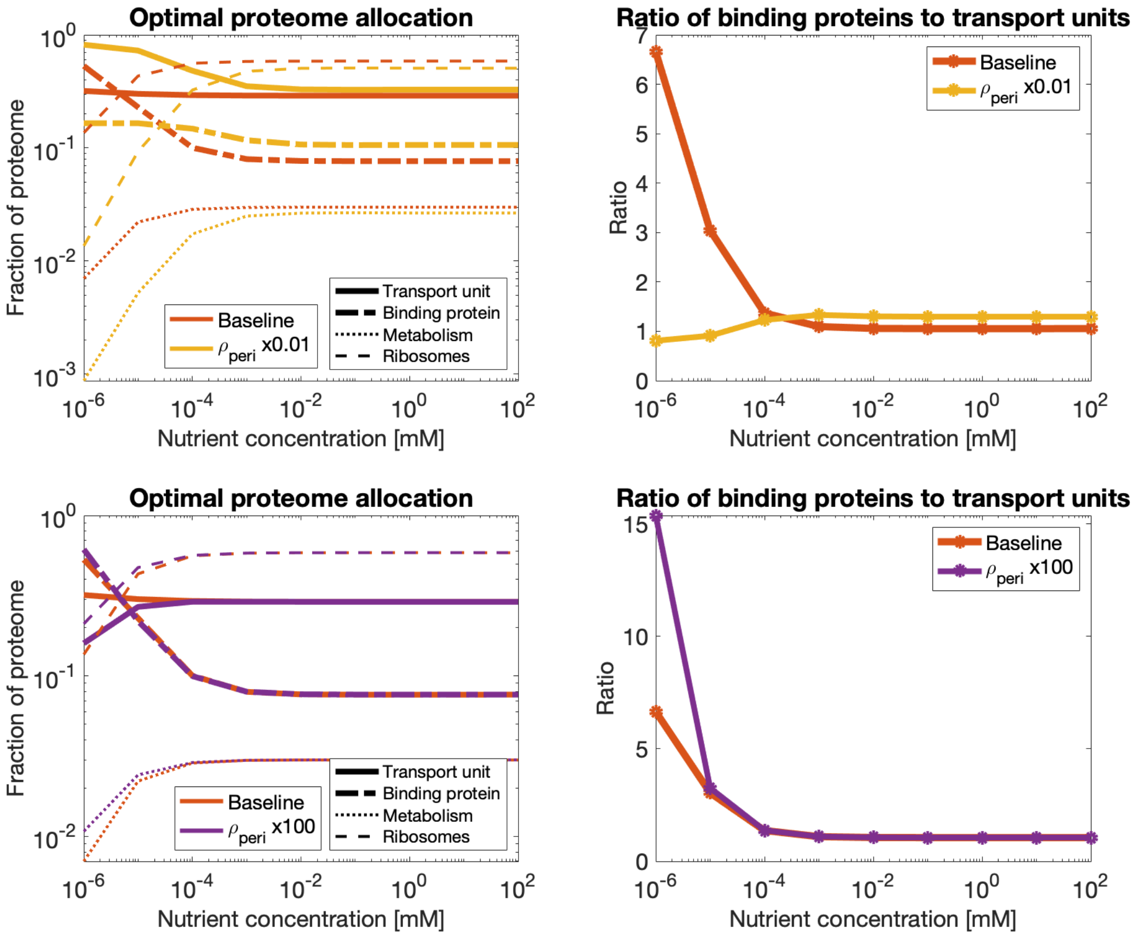

Fig P. Sensitivity analysis, $\rho_{\mathbf{peri}}$: optimal proteome fractions.

B.4 Available surface area, f_{SA}

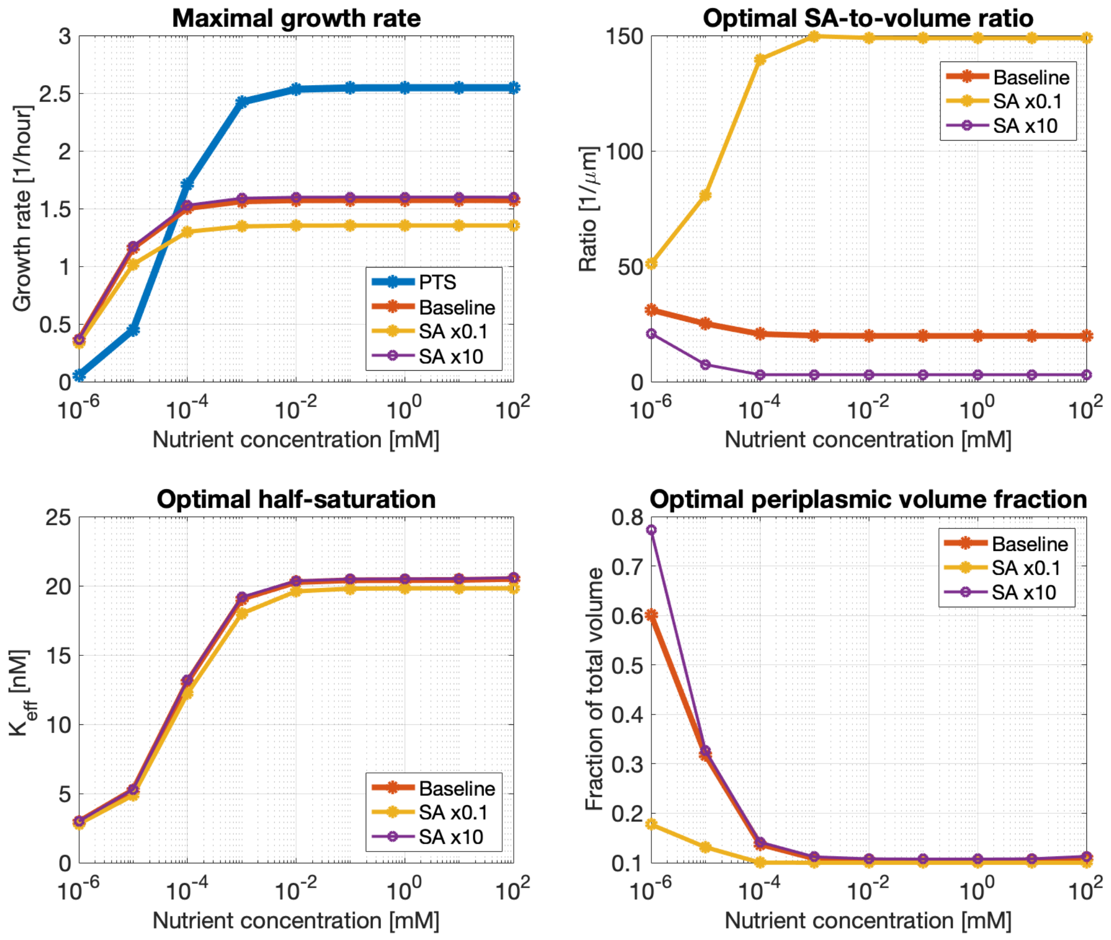

Fig Q. Sensitivity analysis, f_{SA}

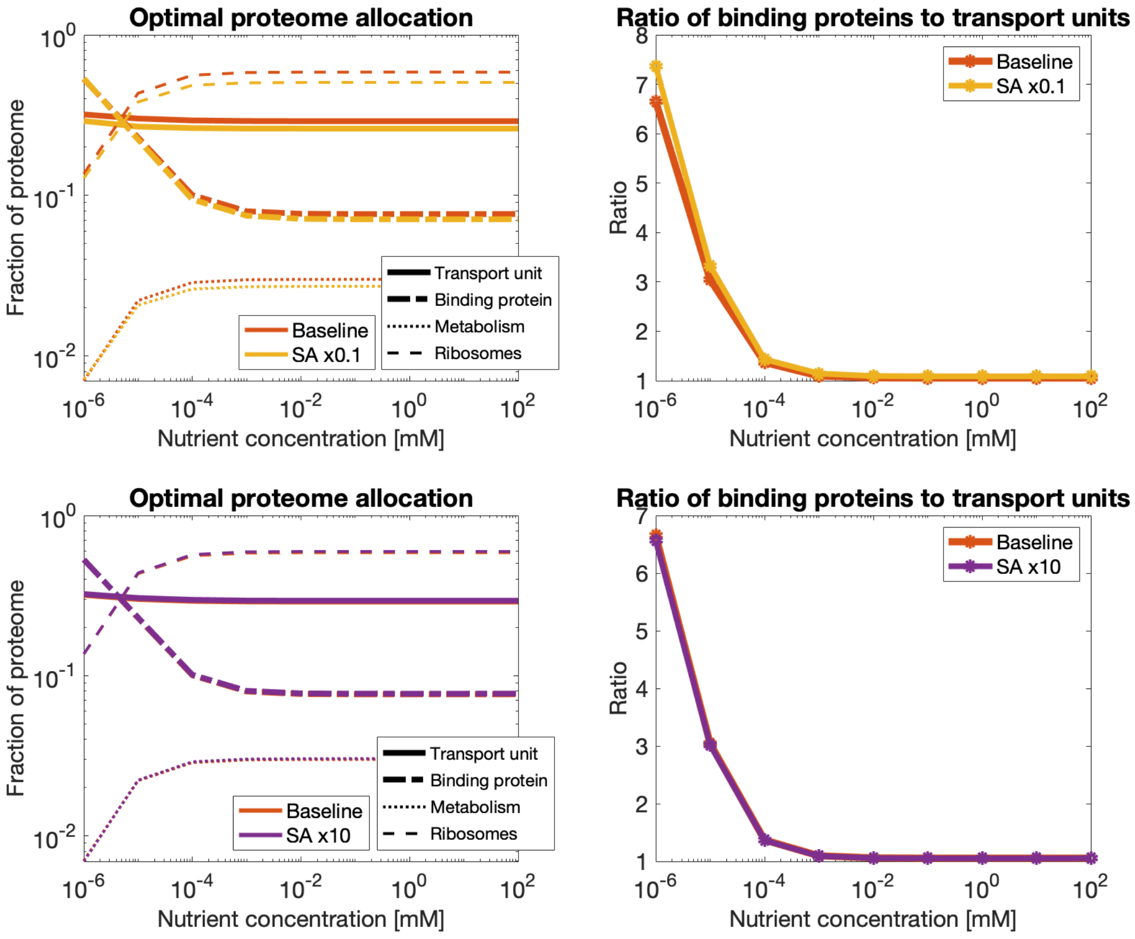

Fig R. Sensitivity analysis, f_{SA}: optimal proteome fractions.

C Binding protein proteomic cost

Here we modified the number of amino acids comprising the binding protein by a factor of 0.1 or 10.

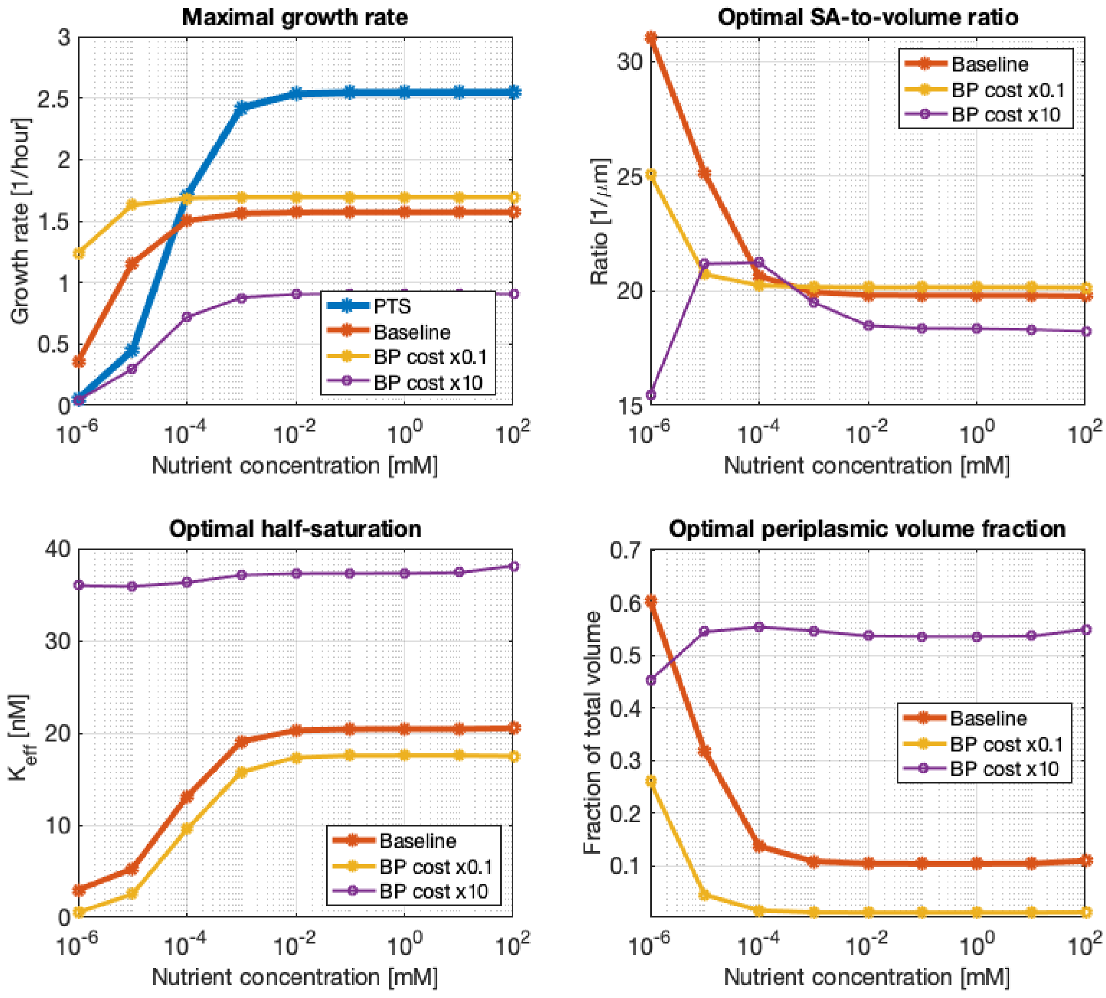

Fig S. Sensitivity analysis, number of amino acids comprising binding protein

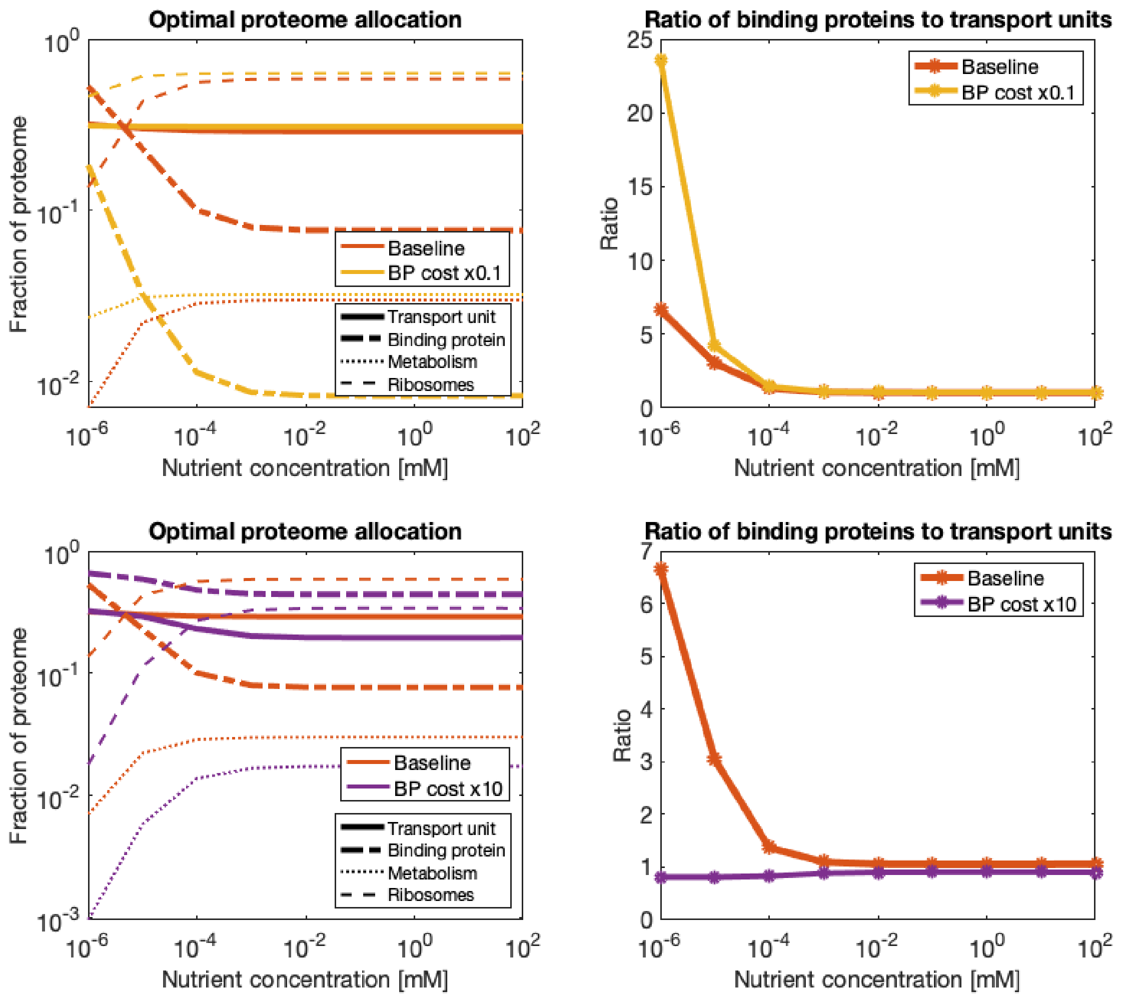

Fig T. Sensitivity analysis,number of amino acids comprising binding protein: optimal proteome fractions.

D Substrate diffusivity

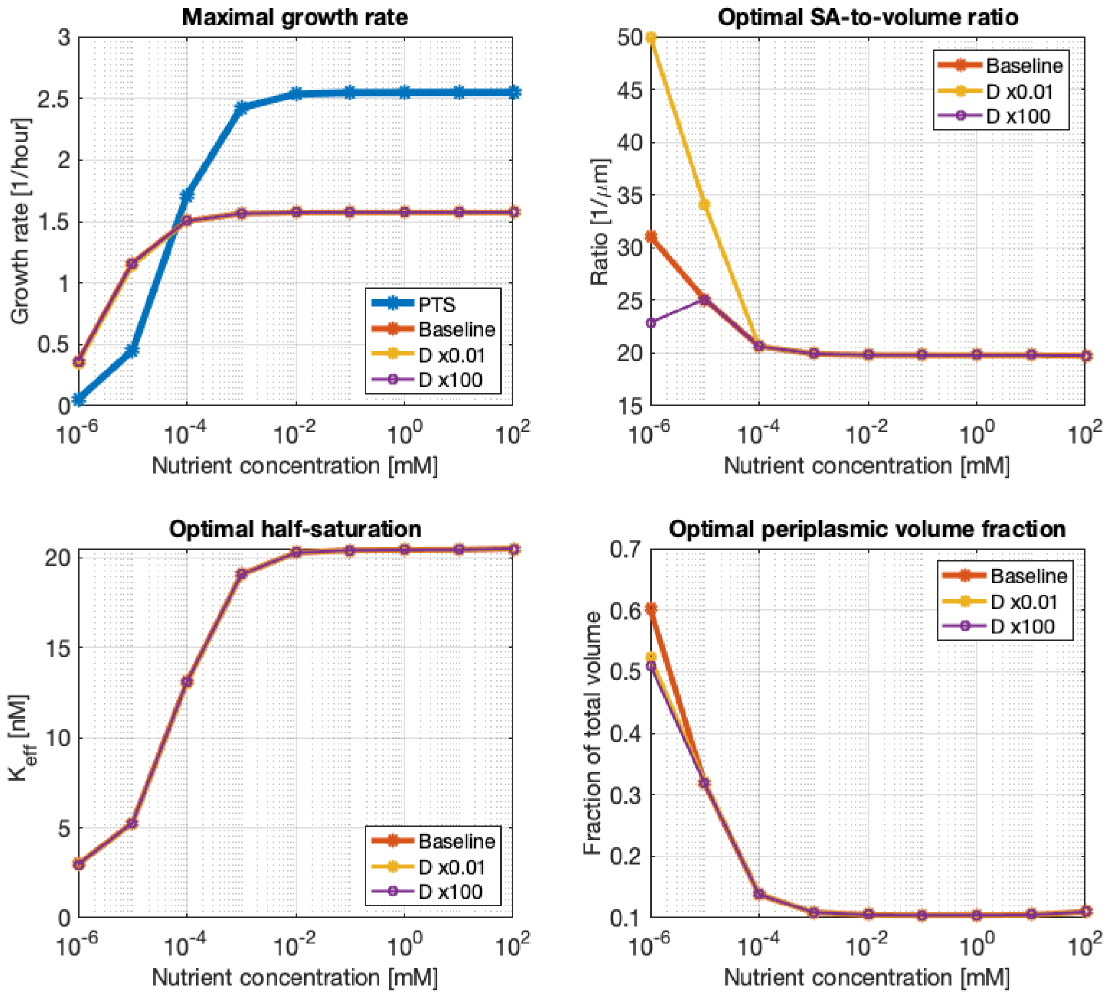

Fig U. Sensitivity analysis, D

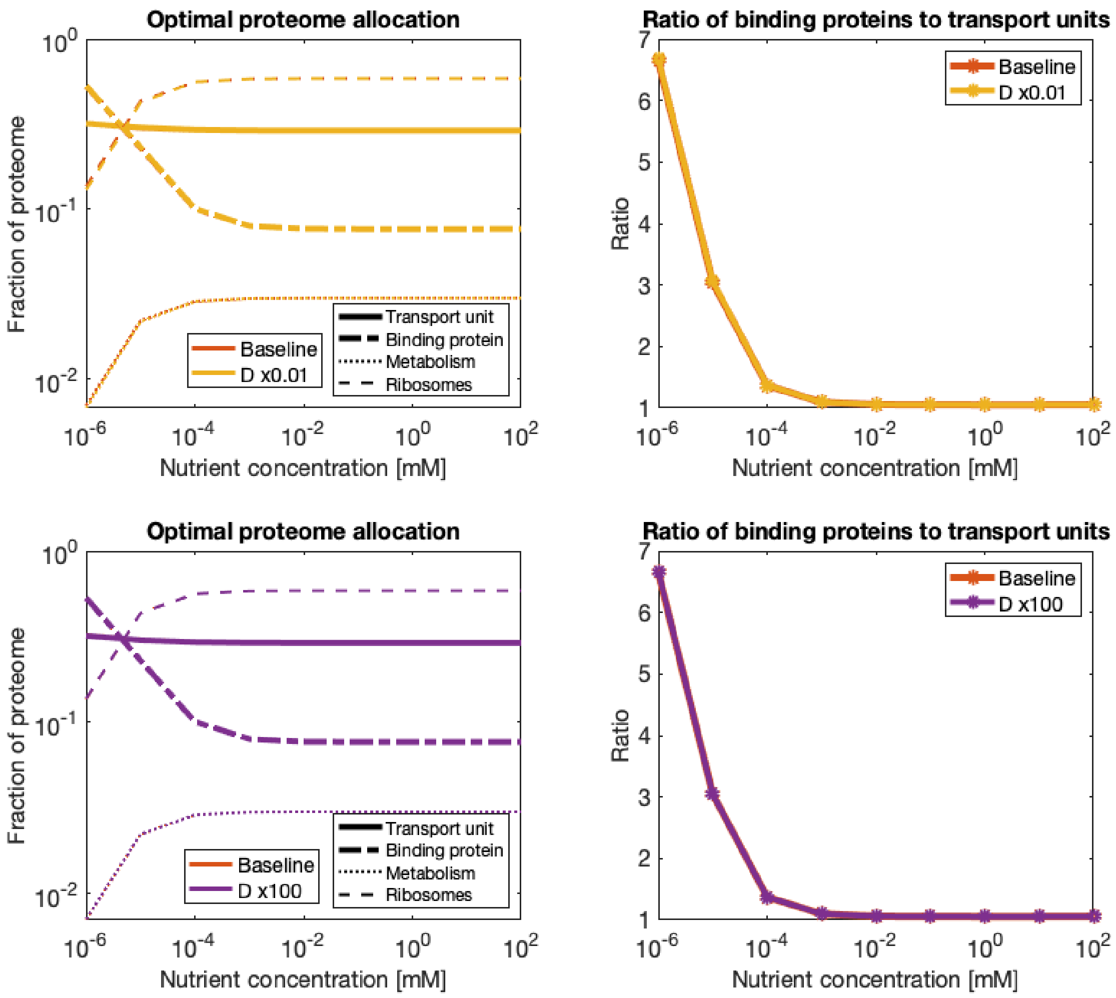

Fig V. Sensitivity analysis, D: optimal proteome fractions.
