# Supplementary material for: Mechanistic model of nutrient uptake explains dichotomy between marine oligotrophic and copiotrophic bacteria
Source: PLoS Comput Biol. 2021 May 19;17(5):e1009023. doi: 10.1371/journal.pcbi.1009023 (PMC8168909; doi:10.1371/journal.pcbi.1009023)
Supplement: S3 Appendix — This supplemental appendix presents Figs A-G, which assesses the sensitivity of the rate-affinity trade-off by contrasting the optimal ABC and PTS cells when particular parameters are modified. Fig A: PTS versus ABC, transport proteomic costs x10. Fig B: PTS versus ABC, protein synthesis proteomic cost x10. Fig C: PTS versus ABC, ∅O,cyto = 0.5. Fig D: PTS versus ABC, ρcyto x0.01. Fig E: PTS versus ABC, ρcyto x100. Fig F: PTS versus ABC, ρperi x0.01. Fig G: PTS versus ABC, ρperi x100 (PDF) [file pcbi.1009023.s003.pdf]

Mechanistic model of nutrient uptake explains dichotomy between marine oligotrophic and copiotrophic bacteria

S3 Appendix: Sensitivity analyses of rate-affinity trade-off

Noele Norris, Naomi M. Levine, Vicente I. Fernandez, Roman Stocker

Code is accessible at: `https://github.com/noelenorris/ABC_proteome_allocation`

Contents

A Transport cost **2**

B Protein synthesis cost **3**

C Proteome fraction devoted to other proteins in the cytoplasm **4**

D Maximal cytoplasmic density **5**

E Maximal periplasmic density **7**

Here we assess the sensitivity of the rate-affinity trade-off when we modify particular constraints.

A Transport cost

Here we increased both the proteomic cost of the transport unit and the proteomic cost of the binding protein by a factor of ten.

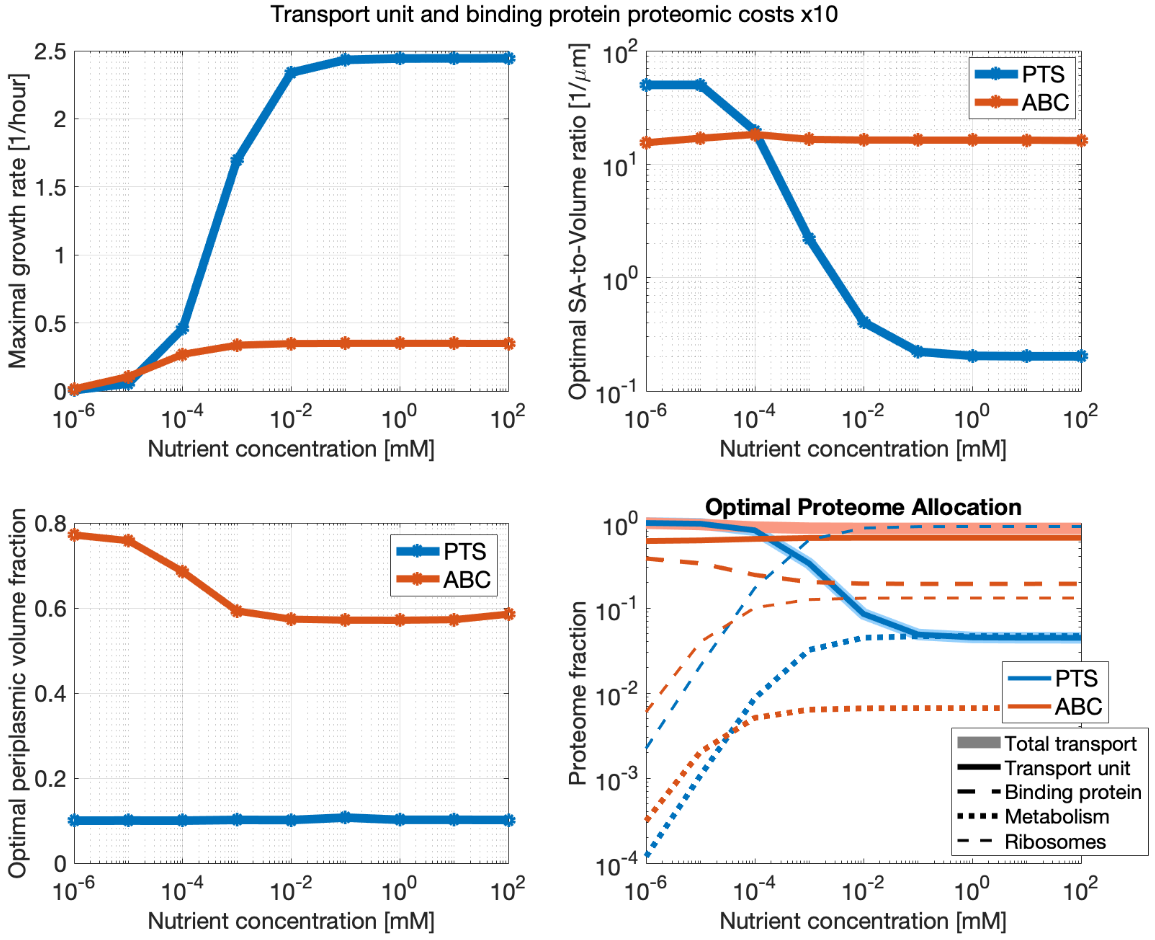

Fig A. PTS versus ABC, transport proteomic costs x10

B Protein synthesis cost

Here we increased the proteomic cost of the ribosomes by a factor of ten.

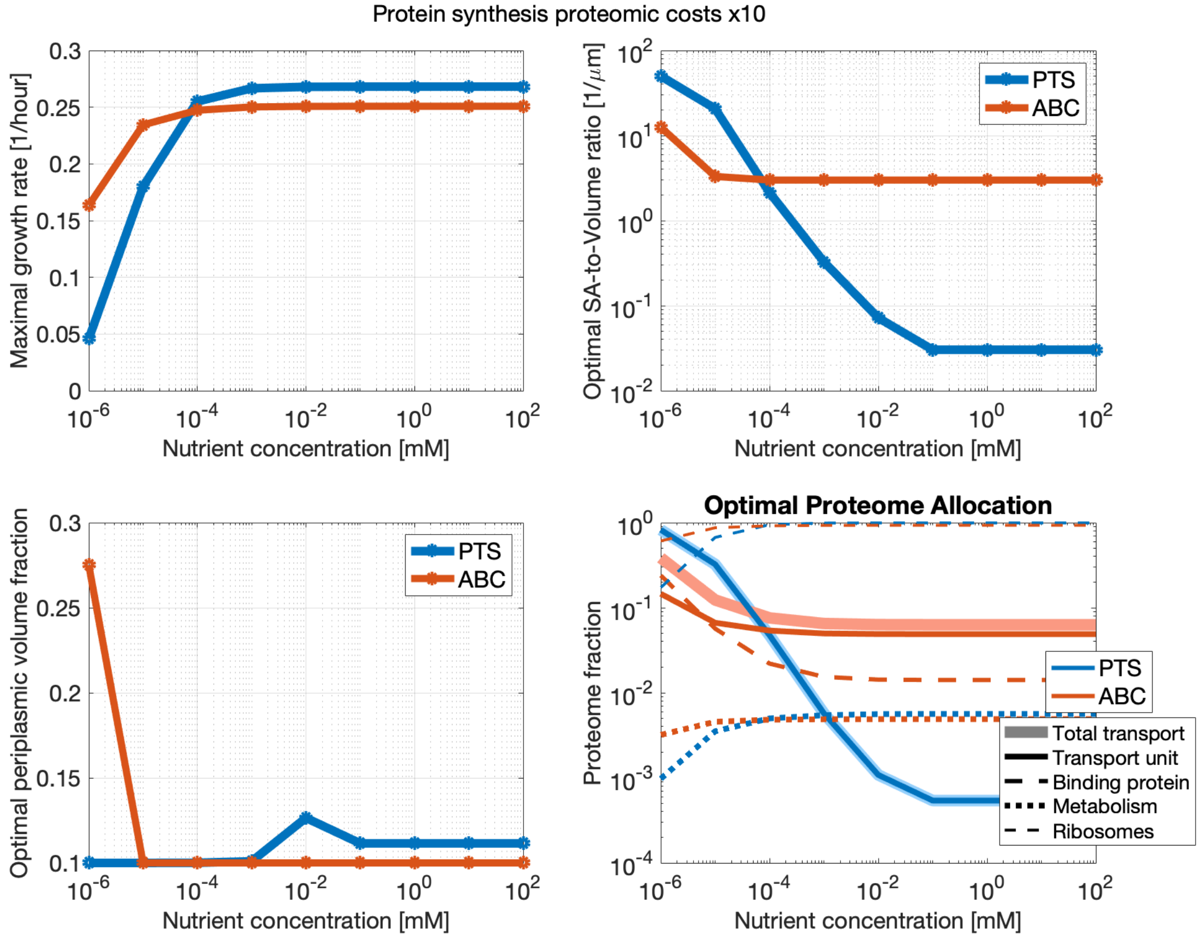

Fig B. PTS versus ABC, protein synthesis proteomic cost x10

C Proteome fraction devoted to other proteins in the cytoplasm

In our baseline case, we allowed the entire proteome be available to the four modeled protein groups, $\phi_{\text{O,cyto}}$. Here we instead take $\phi_{\text{O,cyto}} = 0.5$.

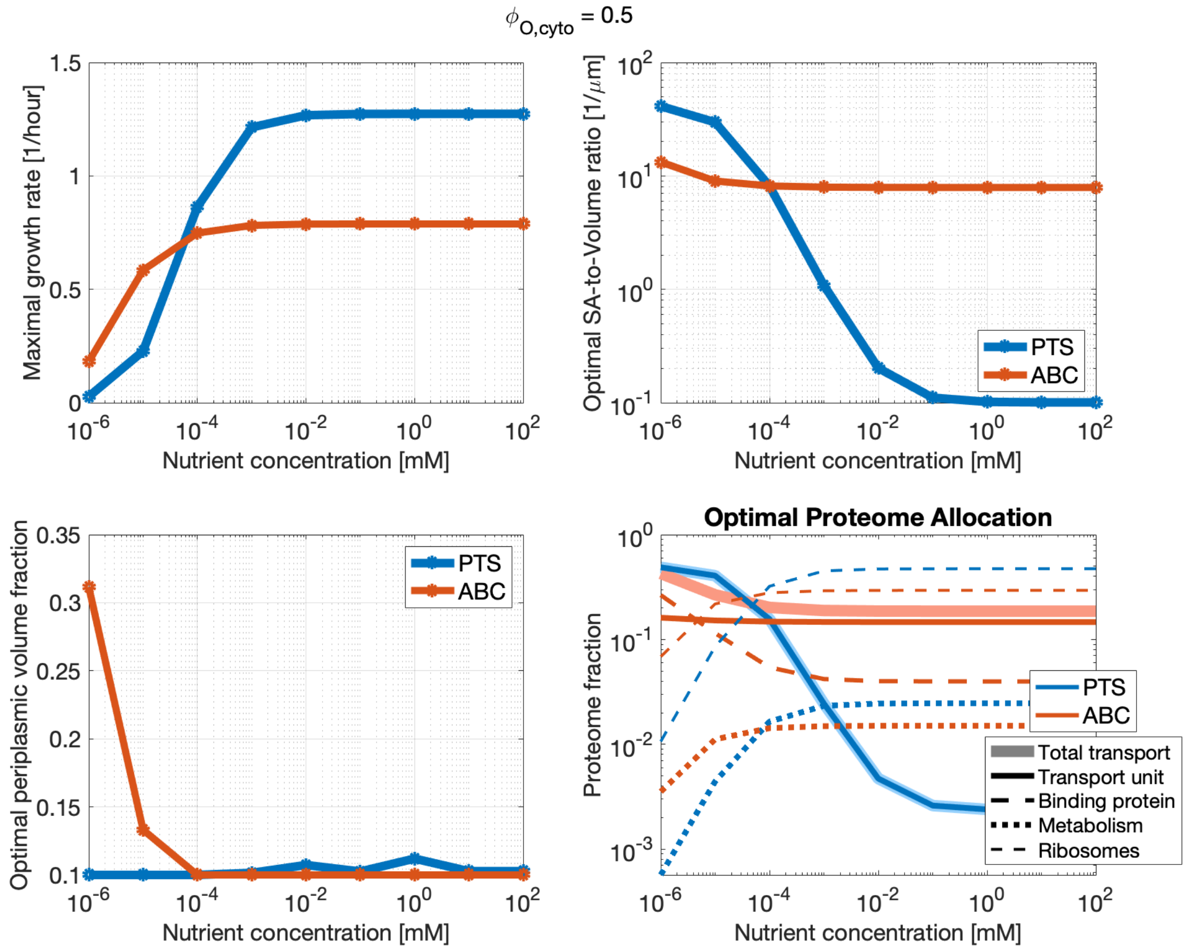

Fig C. PTS versus ABC, $\phi_{\text{O,cyto}} = 0.5$

D Maximal cytoplasmic density

Here we decrease the maximal cytoplasmic density by a factor of 100.

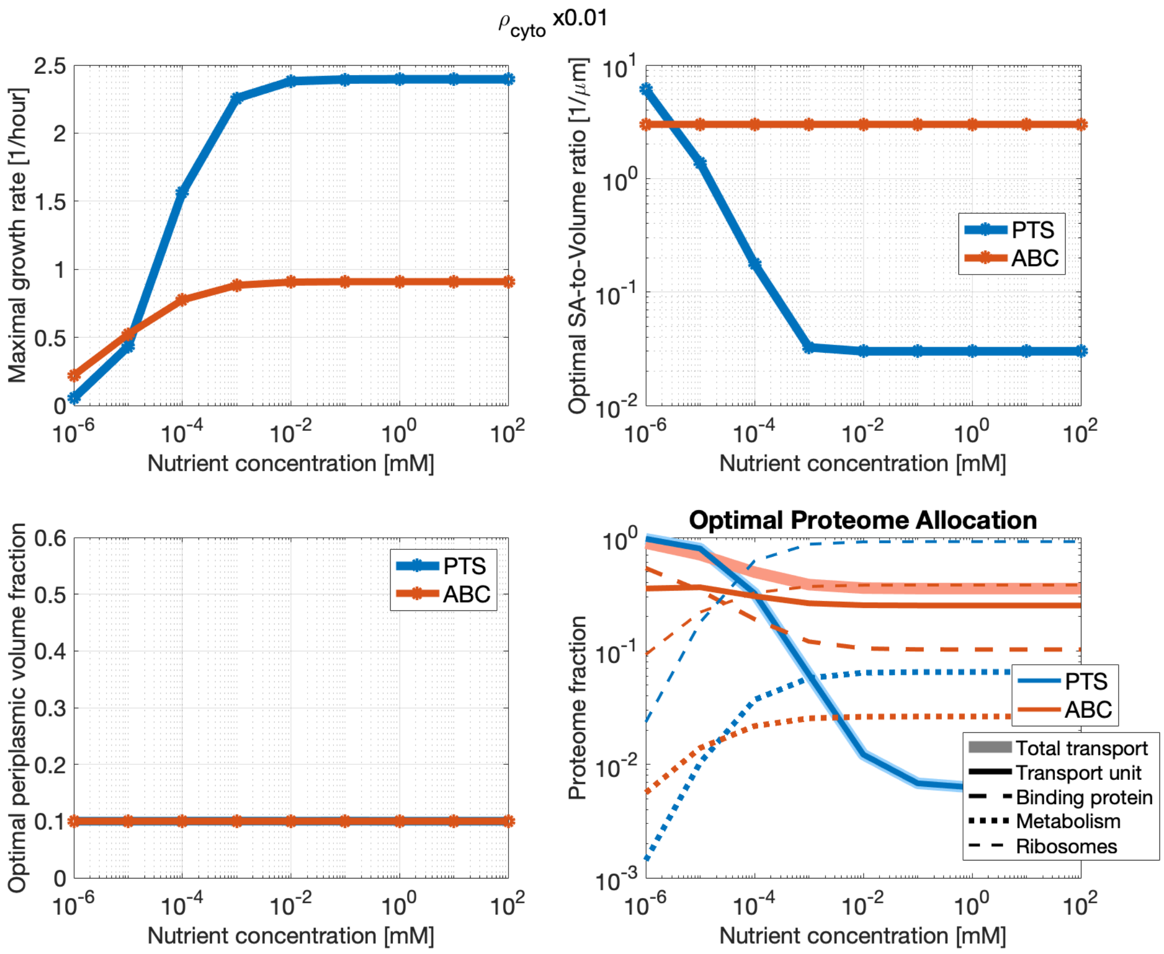

Fig D. PTS versus ABC, $\rho_{\mathbf{cyto}} \times 0.01$

Here we increase the maximal cytoplasmic density by a factor of 100.

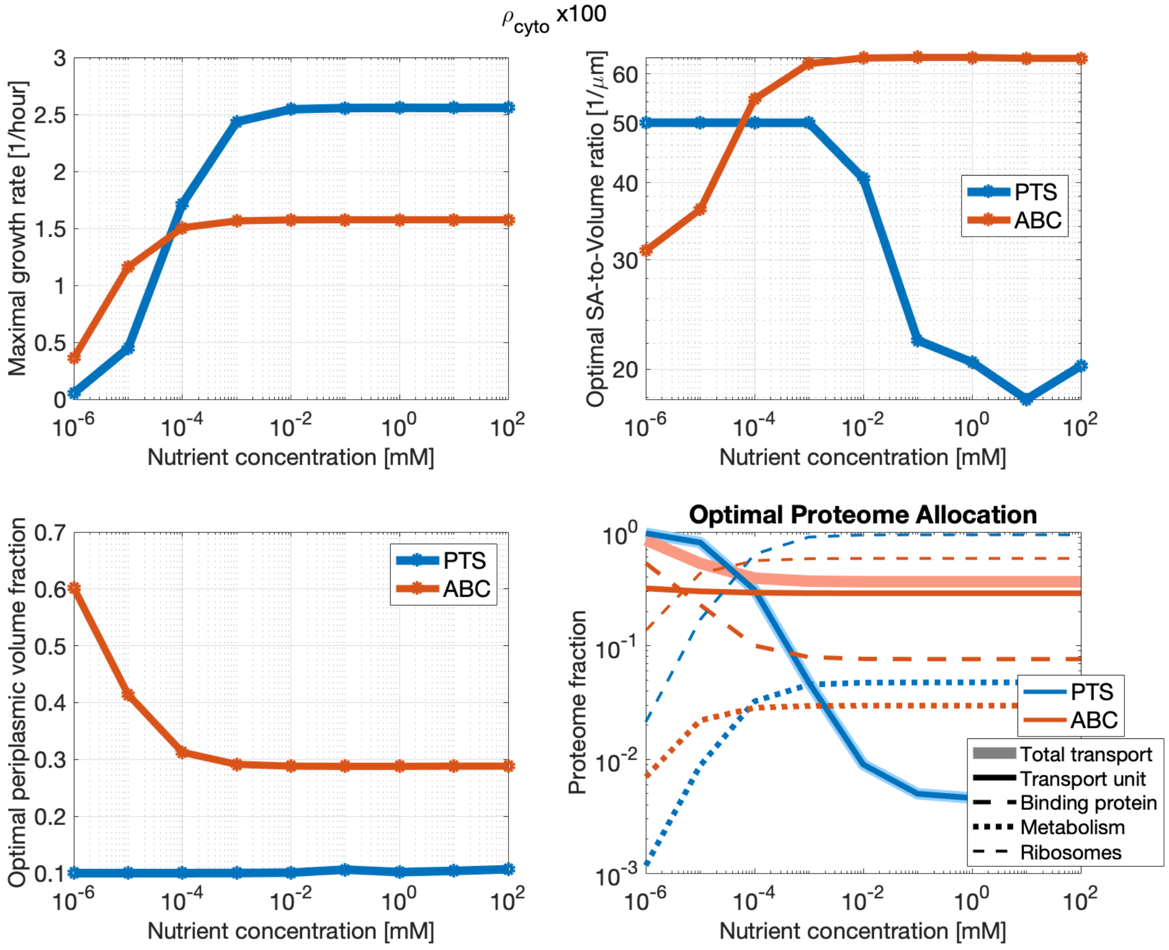

Fig E. PTS versus ABC, $\rho_{\mathbf{cyto}} \times 100$

E Maximal periplasmic density

Here we decrease the maximal periplasmic density by a factor of 100.

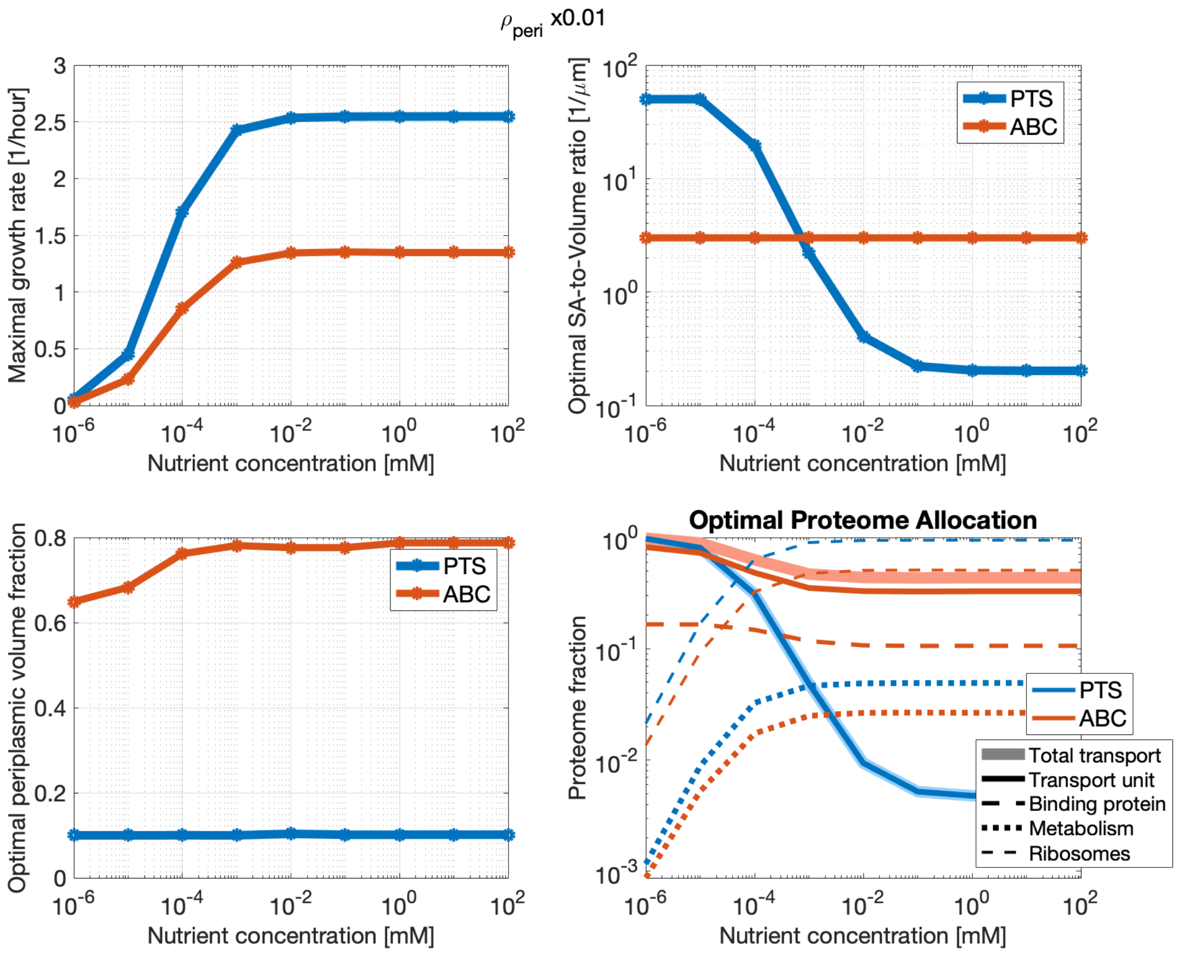

Fig F. PTS versus ABC, $\rho_{\mathbf{peri}} \times 0.01$

Here we increase the maximal periplasmic density by a factor of 100.

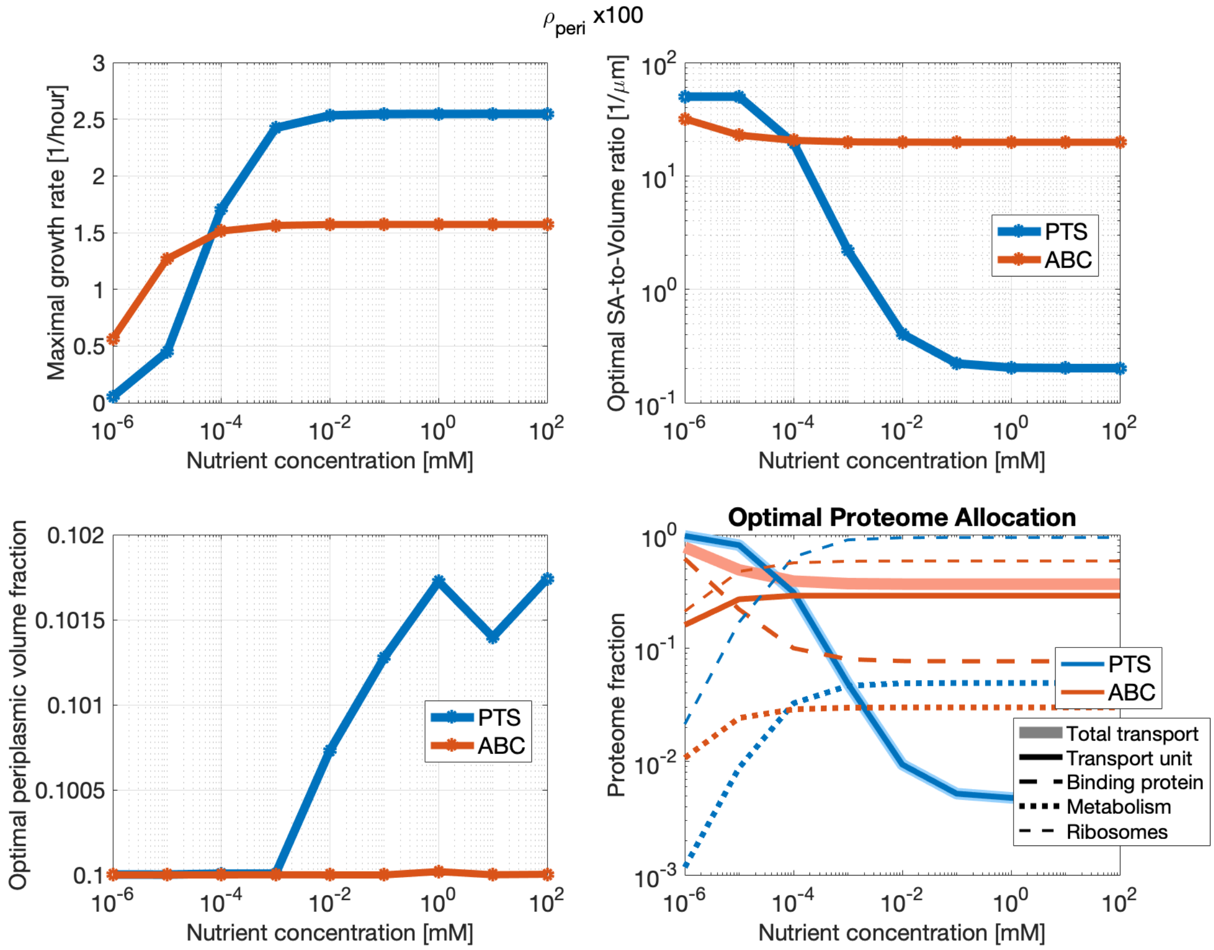

Fig G. PTS versus ABC, $\rho_{\mathbf{peri}} \times 100$
