# Supplementary figures and images for: Mechanistic model of nutrient uptake explains dichotomy between marine oligotrophic and copiotrophic bacteria
Source: PLoS Comput Biol. 2021 May 19;17(5):e1009023. doi: 10.1371/journal.pcbi.1009023 (PMC8168909; doi:10.1371/journal.pcbi.1009023)

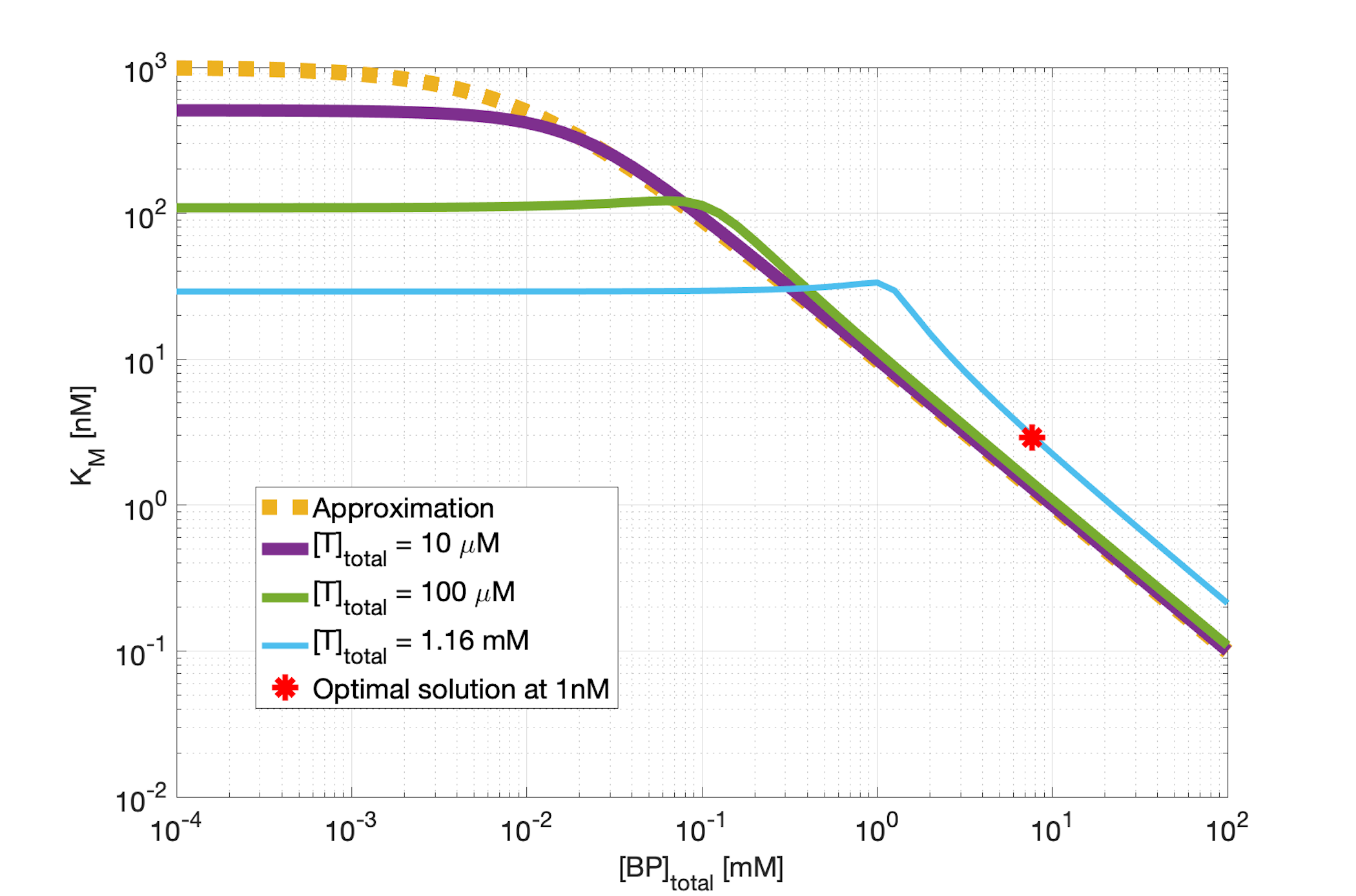

Supplement: S1 Fig — Here we compare our Michaelis-Menten approximation of the half-saturation concentration for ABC transport with the exact half-saturation concentration obtained by solving the set of four equations for ABC transport rates using baseline values for the kinetics rates and modifying the periplasmic concentration of transport units and binding proteins. Note that, for a periplasmic transport unit concentration of 1.16 mM, the half-saturation concentration does not asymptote to the approximation because, in this case, [T]total>k′0r/k′1f. Yet the exact solution follows the same trend as the approximation. (TIF) [file pcbi.1009023.s004.tif]

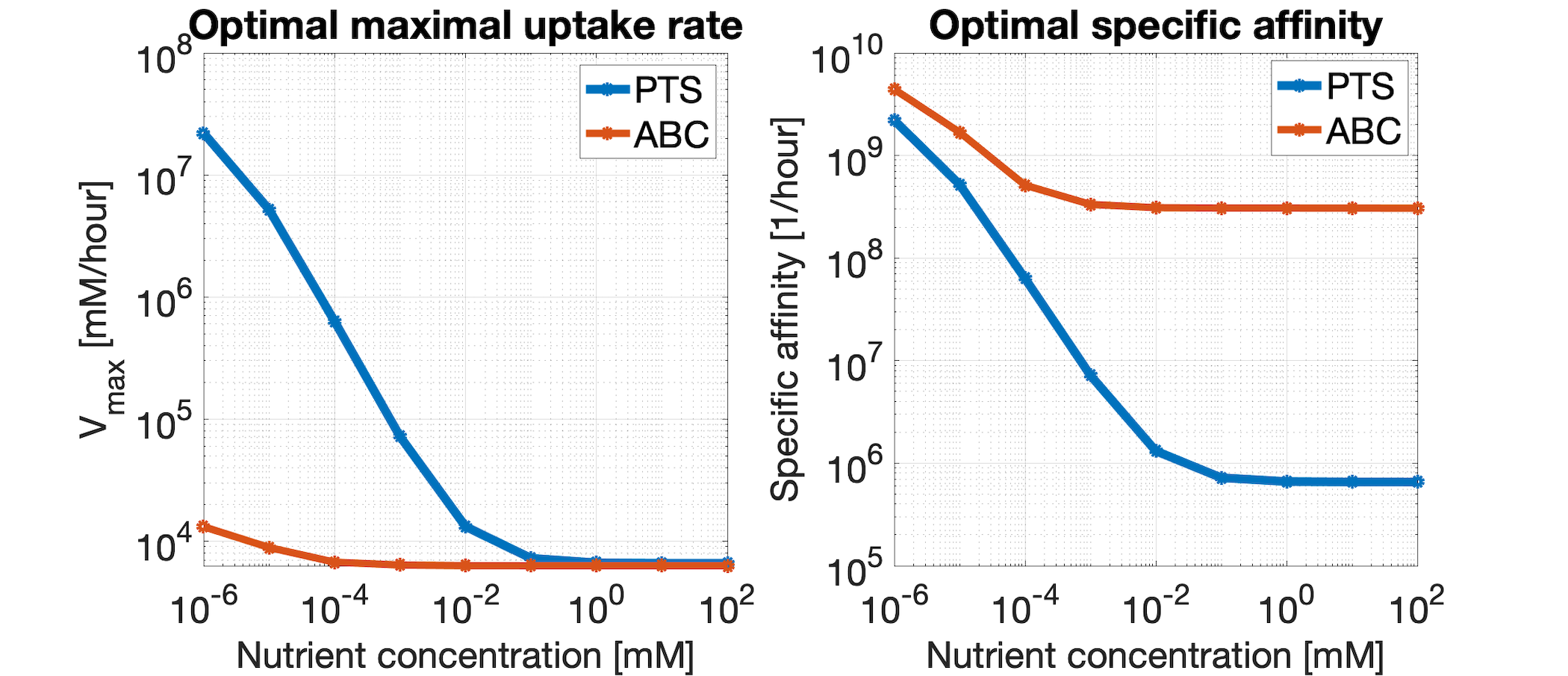

Supplement: S2 Fig — Here are plots showing the optimal maximal uptake rates, Vmax, and corresponding optimal specific affinities, Vmax/KM. (TIF) [file pcbi.1009023.s005.tif]

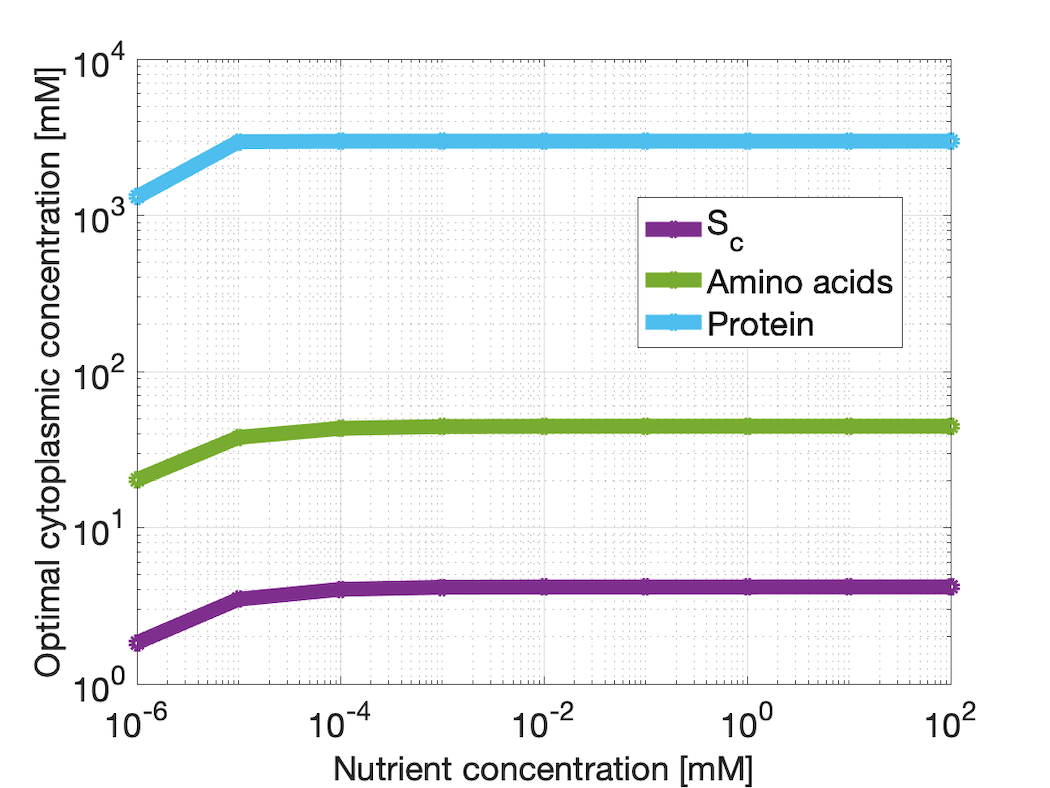

Supplement: S3 Fig — Optimal cytoplasmic concentrations of intracellular nutrient (Sc), amino acids, and total protein (in units of amino acids) over extracellular nutrient condition for cell with ABC transport. Although the extracellular nutrient concentration varies over many magnitudes, the optimal intracellular concentrations vary by less than a factor of three. (TIF) [file pcbi.1009023.s006.tif]

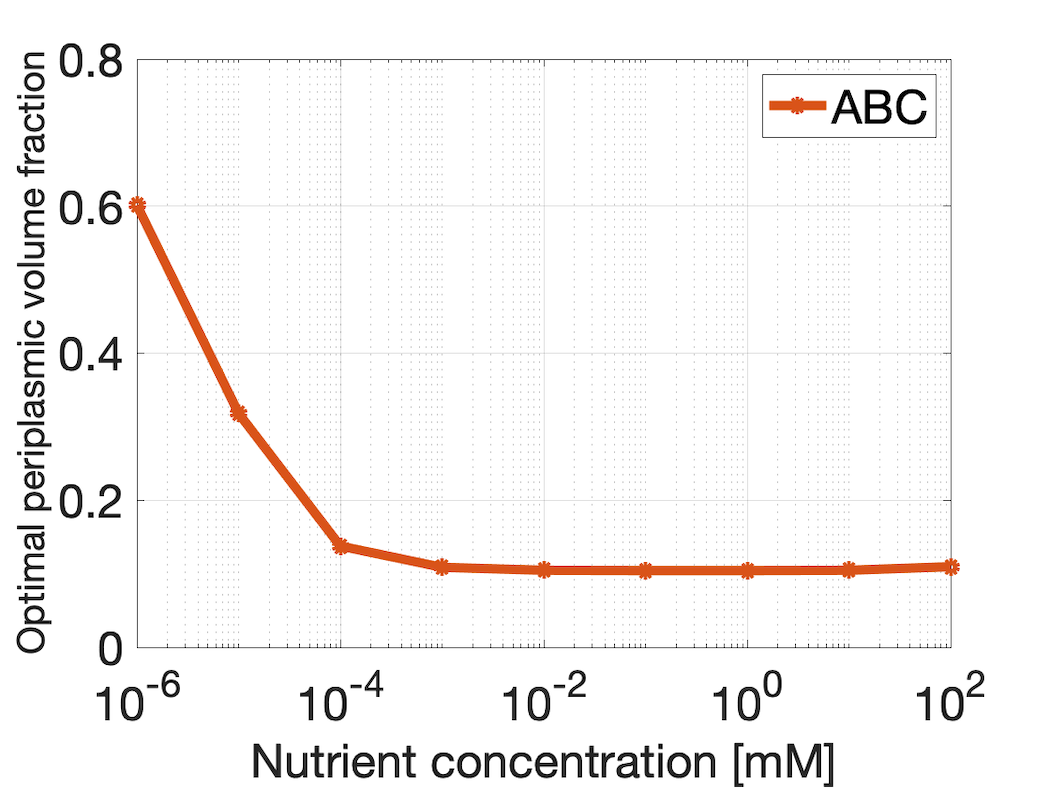

Supplement: S4 Fig — The optimal periplasmic volume fraction increases as nutrient concentration decreases to allow for greater abundances of binding proteins, which are subject to a density constraint on the periplasm. (TIF) [file pcbi.1009023.s007.tif]

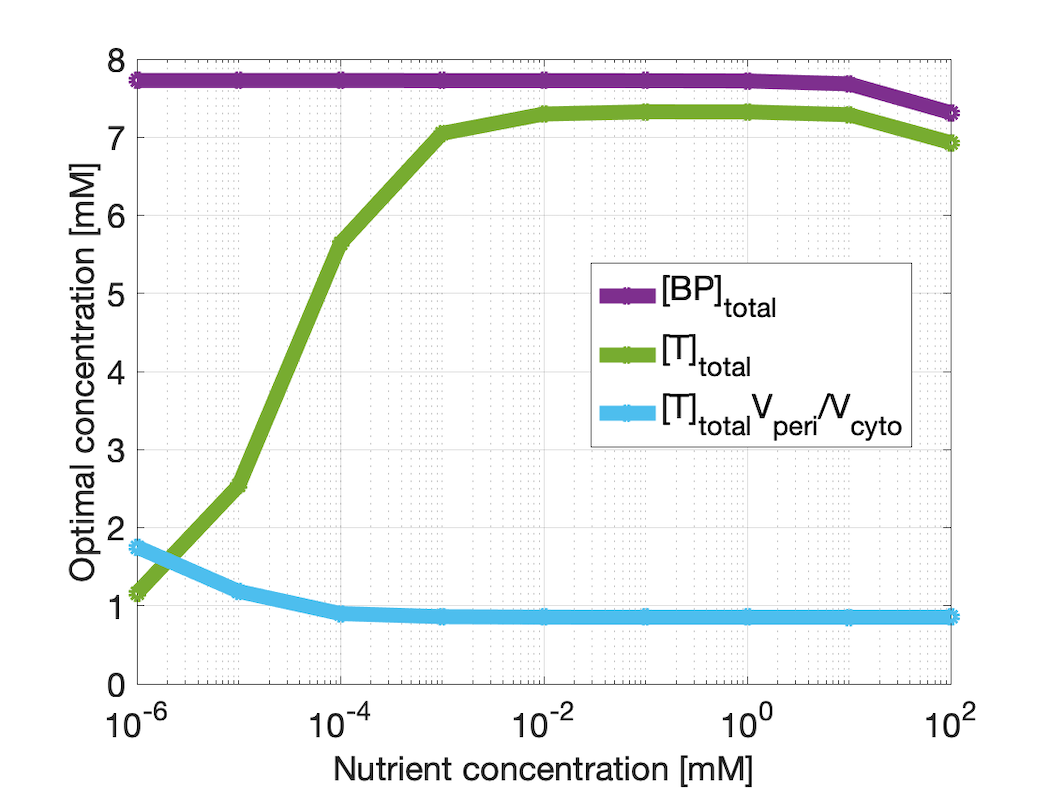

Supplement: S5 Fig — Although the optimal binding protein concentration remains nearly constant over all extracellular nutrient concentrations, the periplasmic transport unit concentration ([T]total) decreases as nutrient concentration decreases due to the inflation of the periplasm. While the periplasm inflates, the cytoplasm shrinks so that, for an extracellular nutrient concentration of 1 nM, the optimal periplasmic concentration of transport units is less than the abundance of transport units divided by the cytoplasmic volume ([T]totalVperi/Vcyto). (TIF) [file pcbi.1009023.s008.tif]

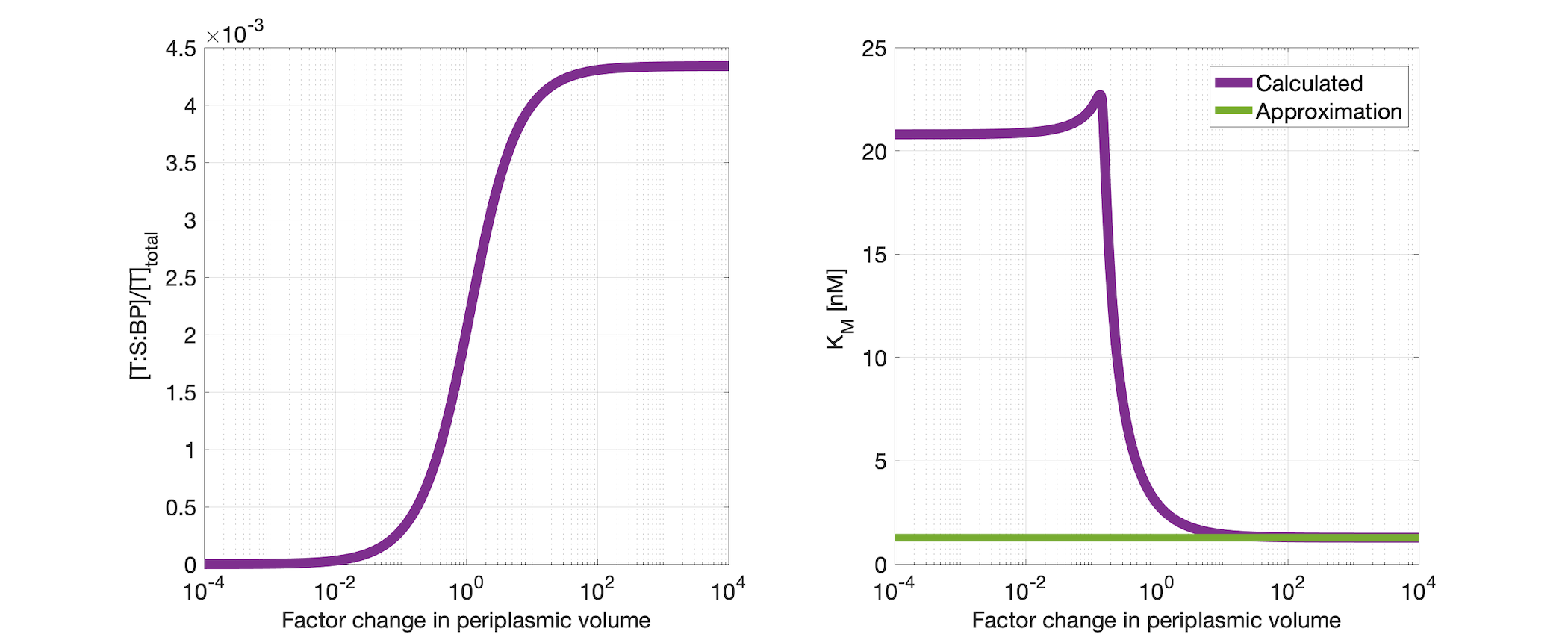

Supplement: S6 Fig — To understand why the periplasm inflates as the nutrient concentration decreases to 1 nM, we plot the proportion of bound transport units (A) and effective half-saturation constant, KM, (B) as we modify the periplasmic volume of the optimal solution for a nutrient concentration of 1 nM. We assumed that both the concentration of binding proteins in the periplasm and the abundance of transport units on the inner membrane remain constant. Therefore, as the periplasm grows, the periplasmic concentration of transport units decreases and the ratio of binding proteins to transport units increases. (A) shows how the increase in abundance of binding proteins due to the inflation of the periplasm leads to an increase in the proportion of bound transport units, where we here assume that the concentration of free substrate in the periplasm ([S]p) is equal to 1 nM. (B) shows the calculated half-saturation constant by fitting the Michaelis-Menten equation to the exact solutions of ABC transport uptake (Eqs 2 to 5), as well as our Michaelis-Menten approximation of the half-saturation constant (Eq 6), which holds only when the binding protein concentration sufficiently exceeds the transport unit concentration. (TIF) [file pcbi.1009023.s009.tif]

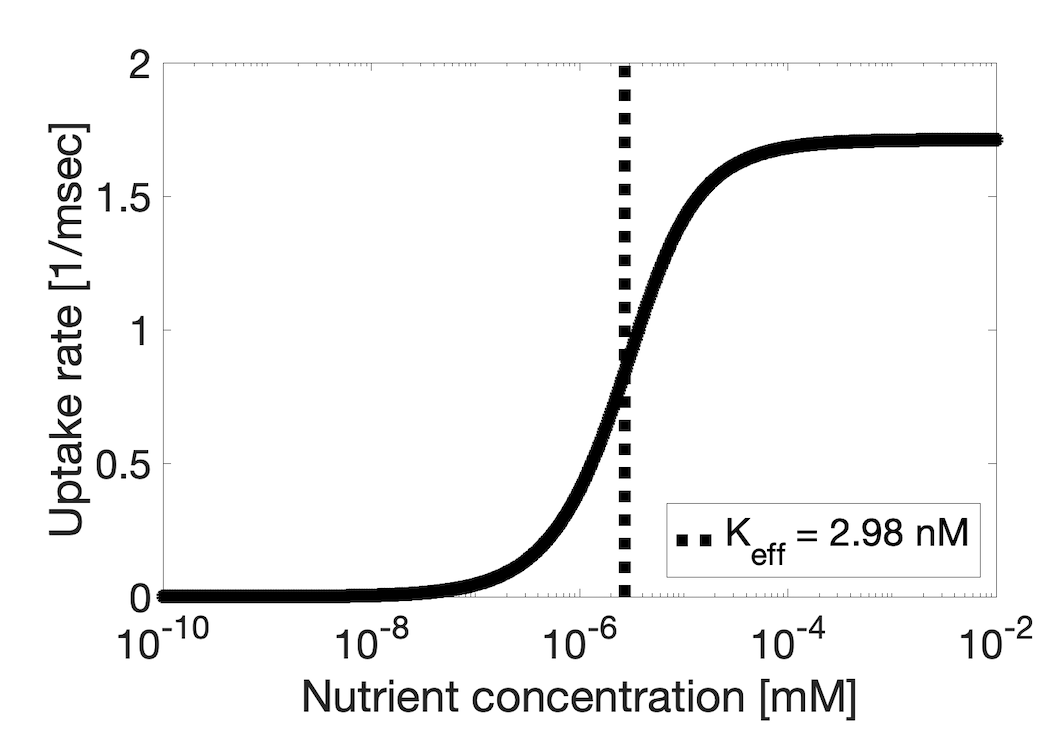

Supplement: S7 Fig — To calculate the effective half-saturation concentration of an optimal solution to a particular proteome allocation problem, we used the system of equations describing ABC transport to determine the uptake rate over a range of nutrient concentrations (x-axis). Here we show the calculated uptake rates over various nutrient concentrations for the proteome allocation obtained when optimized the cell for growth at an extracellular concentration of [S]ext = 1 nM. (TIF) [file pcbi.1009023.s010.tif]

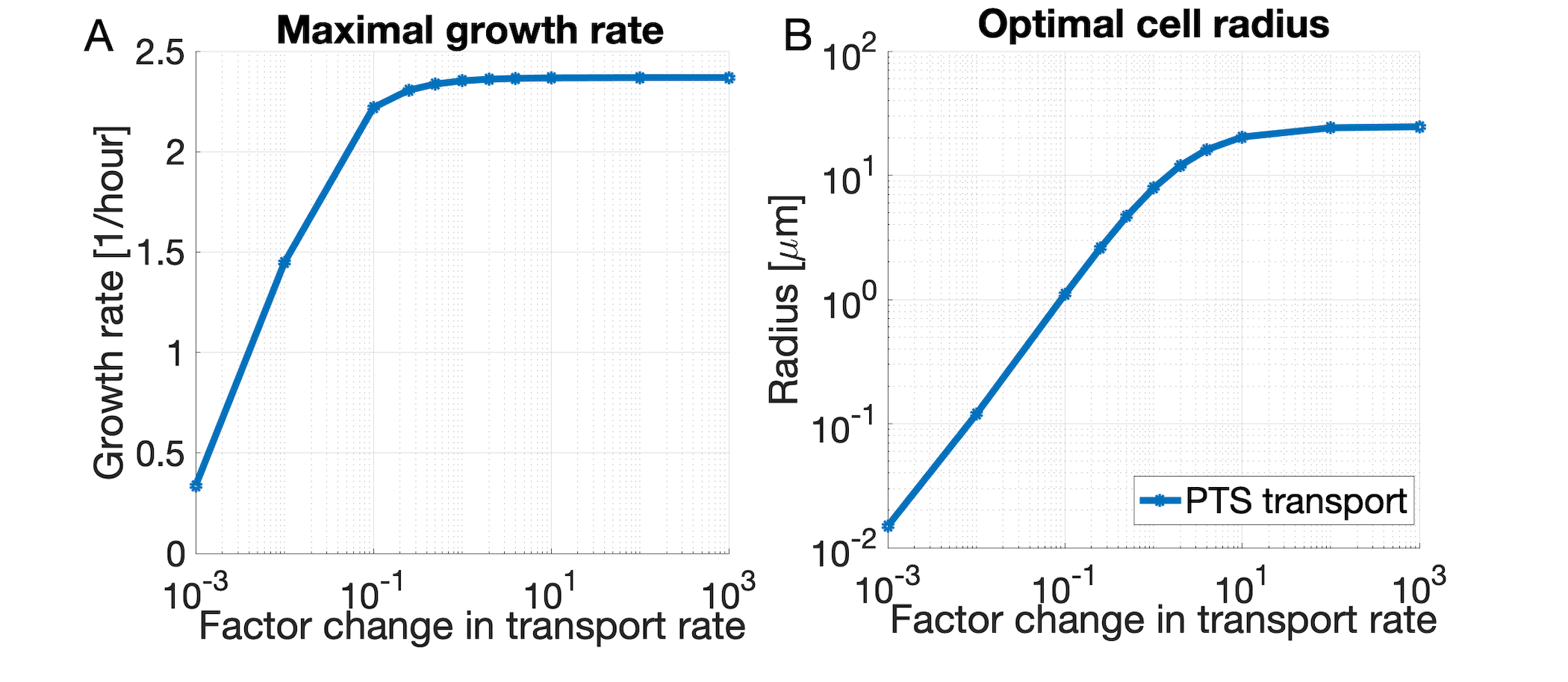

Supplement: S8 Fig — Increases in the translocation rate result in (A) higher achievable growth rates and (B) larger optimal cell radii (that is, smaller surface-area-to-volume ratios). (TIF) [file pcbi.1009023.s011.tif]

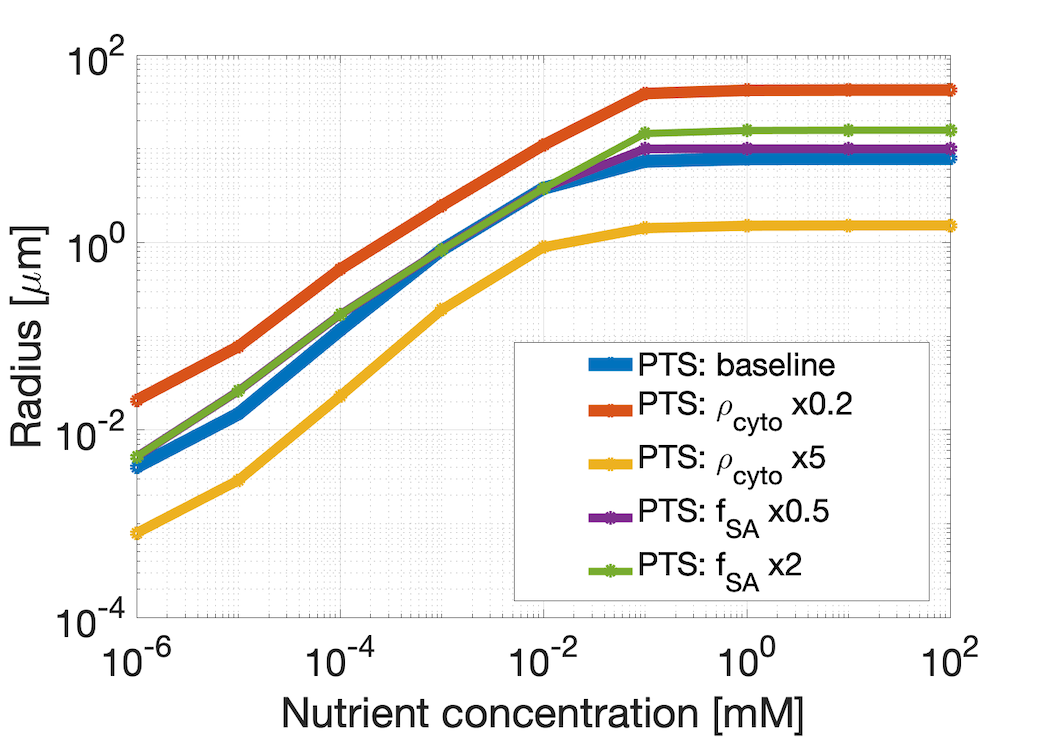

Supplement: S9 Fig — Both the surface area “real estate" constraints and the density constraints are active for the PTS transport proteome allocation problem. Increases in maximal allowed density result in smaller optimal cell radii (red and yellow). Increases in the fraction of the surface area available to the membrane-bound transport units result in larger optimal cell radii (purple and green). (TIF) [file pcbi.1009023.s012.tif]
